# The synthesis of indomethacin prodrugs for the formation of nanosuspensions by emulsion templated freeze drying

**DOI:** 10.1039/d5ra06900a

**Published:** 2025-11-11

**Authors:** Jessica Taylor, Andrew Sharp, Steve P. Rannard, Sarah Arrowsmith, Tom O. McDonald

**Affiliations:** a Department of Chemistry, University of Liverpool Crown Street Liverpool L69 7ZD UK; b Harris-Wellbeing Preterm Birth Research Centre, Department of Women's and Children's Health, Liverpool Women's Hospital, University of Liverpool Crown Street Liverpool L8 7SS UK; c Department of Life Sciences, Manchester Metropolitan University Dalton Building, Chester Street Manchester M1 5GD UK; d Centre of Excellence in Long-acting Therapeutics (CELT), University of Liverpool Liverpool L7 3NY UK; e Department of Materials, Henry Royce Institute, The University of Manchester Manchester M13 9PL UK Thomas.mcdonald@manchester.ac.uk

## Abstract

Emulsion-templated freeze drying (ETFD) is a versatile technique for producing nanosuspensions of poorly water-soluble drugs, but predicting formulation success remains a significant challenge. In this study, we investigate how structural modification of the model drug indomethacin, through esterification with a series of alkyl and aromatic groups, influences nanosuspension formation *via* ETFD. A panel of seven indomethacin prodrugs was synthesised and screened across binary combinations of water-soluble stabilisers. The resulting formulations were assessed based on particle diameter, polydispersity index (PDI), and visual dispersion quality. Analysis of stabiliser combinations revealed specific systems that consistently supported nanoparticle formation across multiple prodrugs. Additionally, there was a positive relationship between increased hydrophobicity, represented by the calculated log *P*, and the formation of viable nanosuspensions. Moreover, the stability of these nanosuspensions was assessed, revealing that esters with higher log *P* values exhibited better dispersion stability. The findings provide valuable insights into the selection of active pharmaceutical ingredients for nanosuspension formulation and further the understanding of the influence of drug properties on nanosuspension stability and production. This research contributes to the development of effective nanosuspension strategies for a wide range of poorly water-soluble pharmaceutical compounds.

## Abstract 2

Increasing log *P* in a rational series of indomethacin prodrugs improves nanosuspension formation by emulsion-templated freeze drying to give stable dispersible dry formulations.

## Graphical abstract

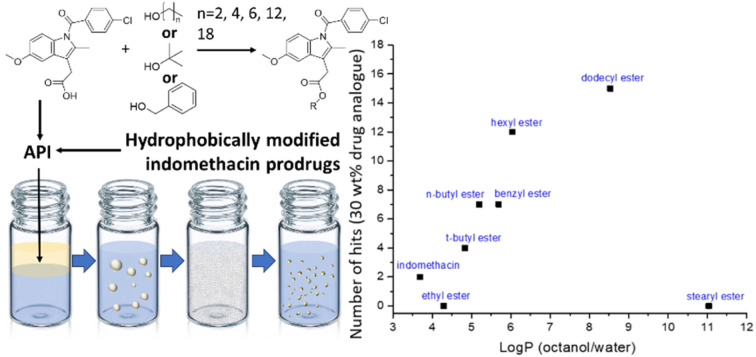

## Introduction

Nanosuspensions are submicron, colloidally stable particles of active pharmaceutical ingredients (APIs) dispersed in a liquid medium, and have emerged as a useful formulation approach for delivering poorly water soluble compounds.^1^ The higher specific surface area of nanosuspensions has been shown to increase the apparent saturation solubility^2^ and the dissolution rate.^3^ These factors can in turn increase the bioavailability of the active pharmaceutical ingredient (API), which is particularly beneficial for compounds with low aqueous solubility.^4^ Nanosuspensions can be produced by various methods,^5^ with the most common including wet milling,^6^ high-pressure homogenisation,^7,8^ nanoprecipitation,^9,10^ and emulsion templated freeze drying (ETFD).^11–17^ The nomenclature used to differentiate nanosuspension is often based on their production method and crystallinity. For example, “nanocrystal” is used to refer to a nanosuspension in which the API is in a crystalline or semi-crystalline state,^6^ while “solid drug nanoparticle” (SDN) is often used for those produced by ETFD.^18^ For the reason of simplicity, the term nanosuspension is used hereafter. The ETFD process involves four stages: (1) production on an emulsion (typically oil-in-water) in which the API is dissolved in a volatile solvent and the aqueous phase contains colloidal stabilisers. (2) Cryogenic freezing of the emulsion. (3) Lyophilisation of the frozen emulsion resulting in a porous monolith. (4) Addition of water which dissolves the porous monolith to release the dispersed nanosuspension.^13^ The mechanism of nanoparticle formation during the ETFD process is not fully understood, but it has been hypothesised that the initial cryogenic freezing causes supercooling of the API solution within the isolated oil droplets resulting in precipitation and/or crystallisation of the API.^11^ Solvent and water removal then prevents the system from redissolving from its kinetically trapped state. The nanoparticles are contained within a matrix of the water-soluble stabilisers which dissolve upon addition of water and release the nanoparticles.^11^ As such, the ETFD process yields dry porous monoliths that can be rapidly redispersed to form the nanosuspension at the time of use. This is an attractive feature as while it is possible to produce redispersible solids of nanosuspensions created by other means, such as spray drying or nanomilled dispersions, ETFD is a single stage process involving simultaneous nanoparticle formation and dry product formation.^10^ The ETFD approach has been used to produce viable formulations from a wide range of APIs, including lopinavir,^19^ maraviroc,^15^ atovaquone,^20^ prodrugs^21^ and even combinations of fluorescent dyes.^22^ Some of these formulations have been shown to increase oral bioavailability^13^ and provide long-acting drug delivery as depot injections.^20,21,23,24^ Despite the opportunities associated with ETFD, the successful production of nanosuspensions using ETFD can be challenging, due to the limited understanding of the factors governing formulation success including the effect of the API's physicochemical characterisations and selection of stabilisers.

ETFD is particularly applicable to a rapid screening approach where binary combinations of water-soluble colloidal stabilisers are tested with a given oil (solvent) and emulsion phase ratio. To identify viable formulation candidates, the products of screening are dispersed and characterised by dynamic light scattering (DLS) and tested against selection criteria such as achieving sub-micron nanoparticle diameters, minimising polydispersity index (PDI) values, ensuring complete dispersion of the freeze-dried monolith, and achieving reproducibility.^14,15,19,20^ These studies have demonstrated the key role of stabiliser combinations in producing viable nanosuspension formulations, with different combinations being identified as being important for different APIs. For example, substituting one of the two stabilisers used in the formulation has been shown to change the dispersion quality (reproducibility, PDI and dispersity) for the HIV drug maraviroc.^15^ One clear factor that can be identified from prior work is that all APIs that have been successfully formulated into nanosuspensions using ETFD tend to have low aqueous solubilities, with the majority reported as ≤0.01 mg mL^−1^.^13,15,19,20^ The role of the physicochemical properties of the API in the successful production of ETFD-based nanosuspensions is a critical knowledge gap.

**Fig. 1:**
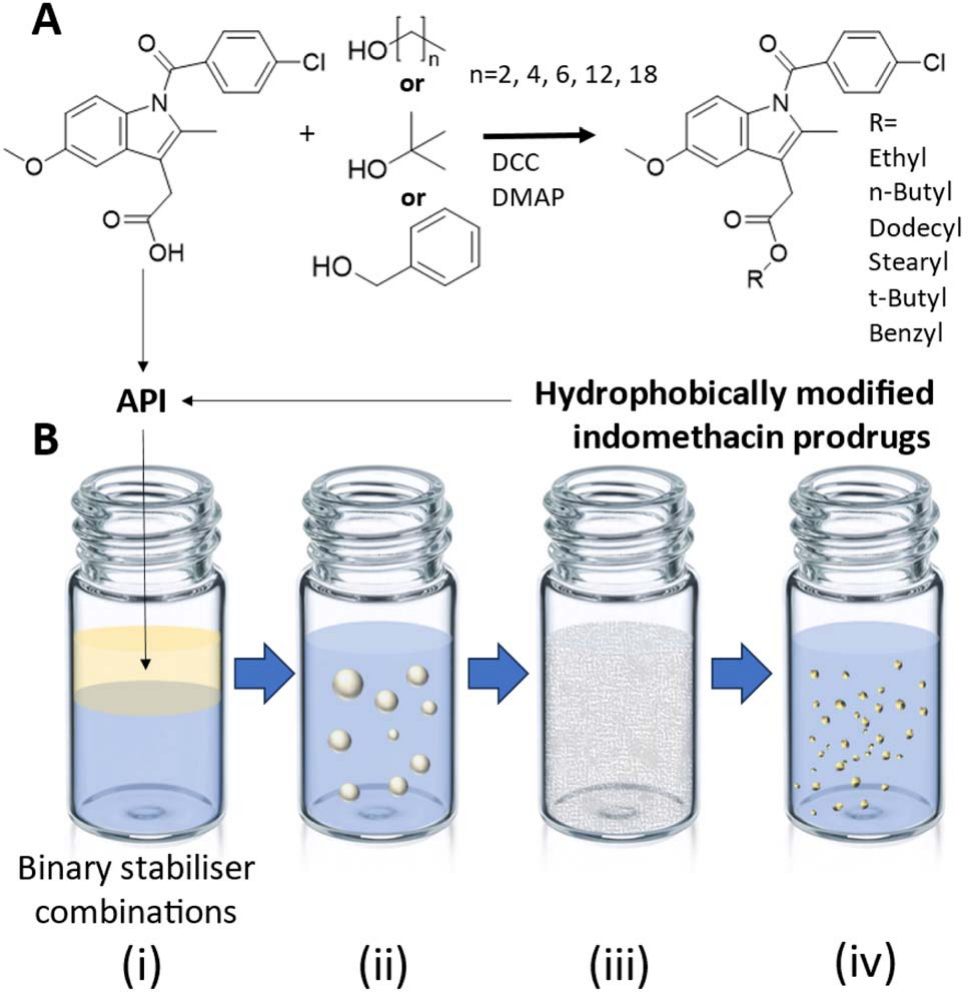
Synthesis of indomethacin prodrugs for the formation of nanosuspensions by emulsion templated freeze drying. (A) Seven hydrophobically modified indomethacin prodrugs were prepared using Steglich esterification on indomethacin using dicyclohexylcarbodiimide (DCC) as a coupling agent and 4-dimethylaminopyridine (DMAP) as a catalyst. Indomethacin or one of the indomethacin prodrugs was used as the API in the ETFD process. 42 different binary combinations of surfactant and polymer stabilisers were screened in the ETFD process. (B) The ETFD process was comprised of four stages: (i) the API was dissolved in chloroform and added to an aqueous solution of stabilisers. (ii) The sample was sonication to produce an emulsion. (iii) The sample was freeze dried resulting in a porous monolith. (iv) The nanosuspension was produced upon the addition of water to the monolith.

Indomethacin, a non-steroidal anti-inflammatory drug (Fig. 1), serves as a model pharmaceutical compound for investigation. It has well-established therapeutic properties and widespread use in treating various inflammatory conditions including the treatment of preterm birth.^25,26^ Indomethacin is a non-polar, hydrophobic molecule as indicated by the log *P* value of 3.69. It also possesses a carboxylic acid group functionality which has a p*K*_a_ value of ∼4.5 (ref. 27) meaning the compound is ionised in pure water. Indomethacin has a relatively low aqueous solubility (0.002 mg mL^−1^ in water and 0.835 mg mL^−1^ in pH 6.8 phosphate buffer),^28^ and is therefore potentially suited to processing as a nanomedicine formulation. The majority of nanomedicine formulations of indomethacin reported are lipid-based, and have been investigated for a range of delivery routes including oral, topical and intravenous, with such systems displaying benefits in terms of improved pharmacokinetics and increased drug accumulation at the target site.^29^ However, there are only a few examples of where indomethacin has been formulated as a nanosuspension, but in these studies the drug loading relative to the inactive ingredients and PDI values were not reported^12,30^ and a centrifuging step was required to remove precipitate from the samples prior to analysis.^30^ As such, it would be beneficial to produce indomethacin nanosuspensions that do not require an additional purification steps that may result in API loss.

Hydrophobic modification of APIs is an effective route to opening up additional formulation options for APIs for polymer nanoparticles,^31^ nanosuspensions^21^ and lipid nanoparticles.^32^ A widely adopted approach involves covalent esterification of APIs with fatty acids, yielding prodrugs with increased lipophilicity which when used to produce nanosuspensions, alter the behaviour of an API.^33^ Despite growing interest in ester prodrugs, there is limited systematic understanding of how structural variation such as alkyl chain length or log *P* affects nanosuspension formation, which in turn, hampers the formulation of these novel therapeutics.

Here, we explore the use of the ETFD process for the production of indomethacin nanosuspensions. In addition to investigating the parent drug, we synthesised a series of seven hydrophobically modified indomethacin ester prodrugs with systematically varied alkyl chain lengths and log *P* values (Fig. 1A). These prodrugs served as a series to examine how changes in molecular structure and log *P* affect formulation behaviour. By conducting a comparative formulation study across multiple binary stabiliser combinations using the EFTD process (Fig. 1B), we aimed to understand how increasing lipophilicity and subtle structural variations influence the likelihood of successful nanosuspension formation *via* ETFD. Our findings show that the most hydrophobic derivatives, particularly those with linear C6 and C12 chains, significantly increased the number of viable formulations across a broader range of stabiliser systems. This advances the formulation of indomethacin-based nanosuspensions and offers new insights into the interplay between molecular structure and formulation outcomes.

## Results and discussion

### Indomethacin nanosuspensions

**Fig. 2:**
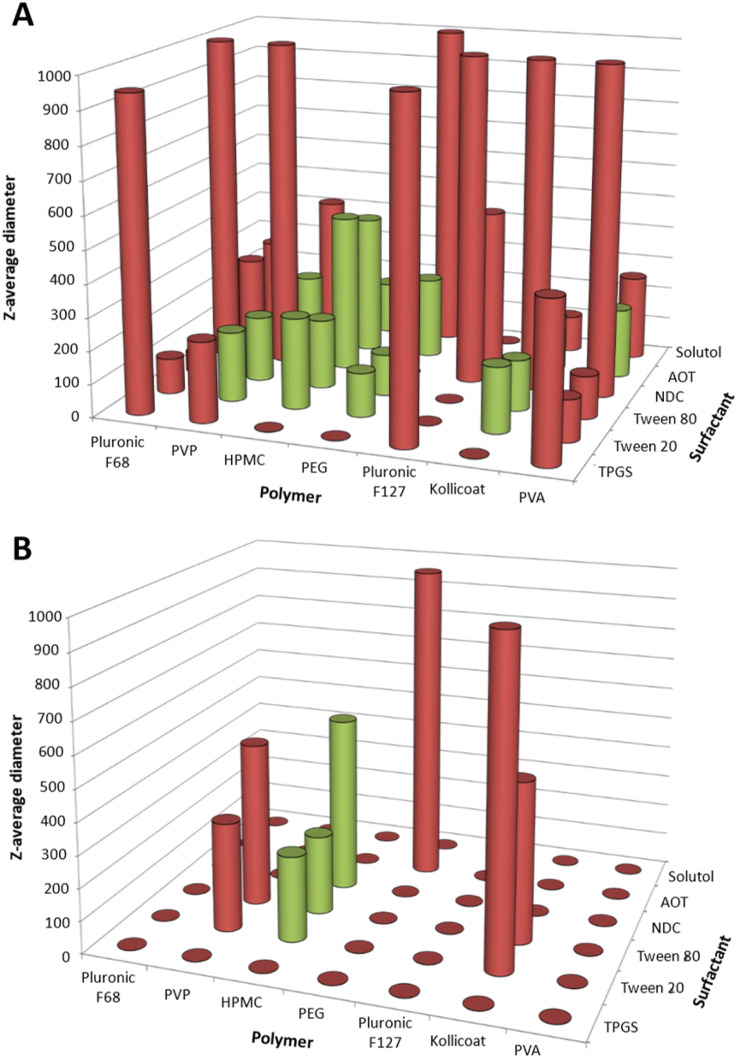
Screening indomethacin nanosuspension formulations by the ETFD method using binary combinations of stabilisers. (A) Summary of the data obtained for 10 wt% indomethacin loading formulations, using assessment criteria of mean diameter <500 nm, a standard deviation of <10% between the triplicate diameter measurements, the absence of any visible particles in the dispersion, and a PDI <0.3. (B) Summary of the data obtained for 30 wt% indomethacin loading formulations, using assessment criteria of a mean diameter of <600 nm, a standard deviation <10%, the absence of any visible particles in the dispersion and a PDI <0.5. In both cases, the polymeric type stabilisers are found on the *x*-axis and the surfactant type stabilisers are those on the *z*-axis. Samples that met the screening criteria are shown in green, samples that did not fully disperse or had a diameter of >1 μm are represented as red circles and unsuccessful samples in red (*i.e.* where the diameter or PDI were above the specified assessment criteria, or standard deviation between the triplicate diameter measurements was of >10%). The full names and structures of the different stabilisers are shown in Fig. S1.

To assess the suitability of ETFD for formulating indomethacin, a binary screen of different stabilisers was investigated with a drug loading of 10% wt loading in the final formulation, *i.e.* 10 mg of the lyophilised total formulation would contain 1 mg of the API. A 10% wt loading was selected for the first screen as this loading has previously been shown for EFFD formulation identification.^19^ The screen involved 13 different stabilisers that were broadly differentiated into six surfactants and seven polymeric stabilisers, resulting in 42 different binary combinations. All of the stabilisers were selected from the U.S. Food and Drug Administration (FDA) list of inactive ingredients for approved drug products (https://www.accessdata.fda.gov/scripts/cder/iig/index.cfm) and the selection was based on those previously reported in ETFD nanosuspension production.^13,19^ It has previously been reported that the lower molecular weight of surfactant type stabilisers can reduce the interfacial tension between the oil and water in the emulsion,^12^ while it is likely that the polymeric stabilisers provide enhanced steric stabilisation. Therefore, polymer stabilisers were only used in combination with the stabilisers listed as surfactants. The structures of all the stabilisers are shown in SI Fig. S1. The mass ratio of the polymer stabiliser to the surfactant type stabiliser was 2 : 1, as this composition has been commonly used in prior publications,^13,14^ making the total mass composition of the total formulation 10% indomethacin, 60% polymeric stabiliser and 30% surfactant stabiliser. The 42 formulations were prepared in triplicate and after freeze-drying all the formulations were assessed for their ability to redisperse in water and analysed by DLS. The viability of each formulation was assessed based on the mean diameter (Z-average) and PDI. Formulations were then deemed viable if they had a mean diameter <500 nm, a standard deviation of <10% between the triplicate diameter measurements, the absence of any visible particles in the dispersion, and a PDI <0.3. These criteria were selected to obtain reproducible, nanosuspensions that could be easily redispersed. PDI values of <0.3 were selected as values below this indicated that the nanoformulation as a homogenous population.^34^ While the <500 nm diameter was chosen as reduction of drug particle diameter increases the effective surface area the dissolution rate.^35^ The results of this analysis are shown in Fig. 2A, with the ETFD formulation of indomethacin producing 14 viable formulations. The polymeric stabilisers appeared to have a larger influence on the formation of viable formulations than the surfactant type, with the most successful formulation found when hydroxypropyl methylcellulose (HPMC), polyvinylpyrrolidone (PVP-K30) or poly(ethylene glycol) (PEG 1 K) were used. This effect was likely due to, in part, there being twice the concentration (mg mL^−1^) of polymeric stabiliser with represent to the surfactant stabiliser. Interestingly, we did not identify poly(vinyl alcohol) (PVA) as a favourable stabiliser despite it being used in previous indomethacin ETFD nanosuspension formulations,^12^ however, this disparity is likely due to the stricter assessment criteria being applied within our study for identifying viable formulations as well as differences in formulation (the solvent and the oil to water phase ratio).

**Fig. 3:**
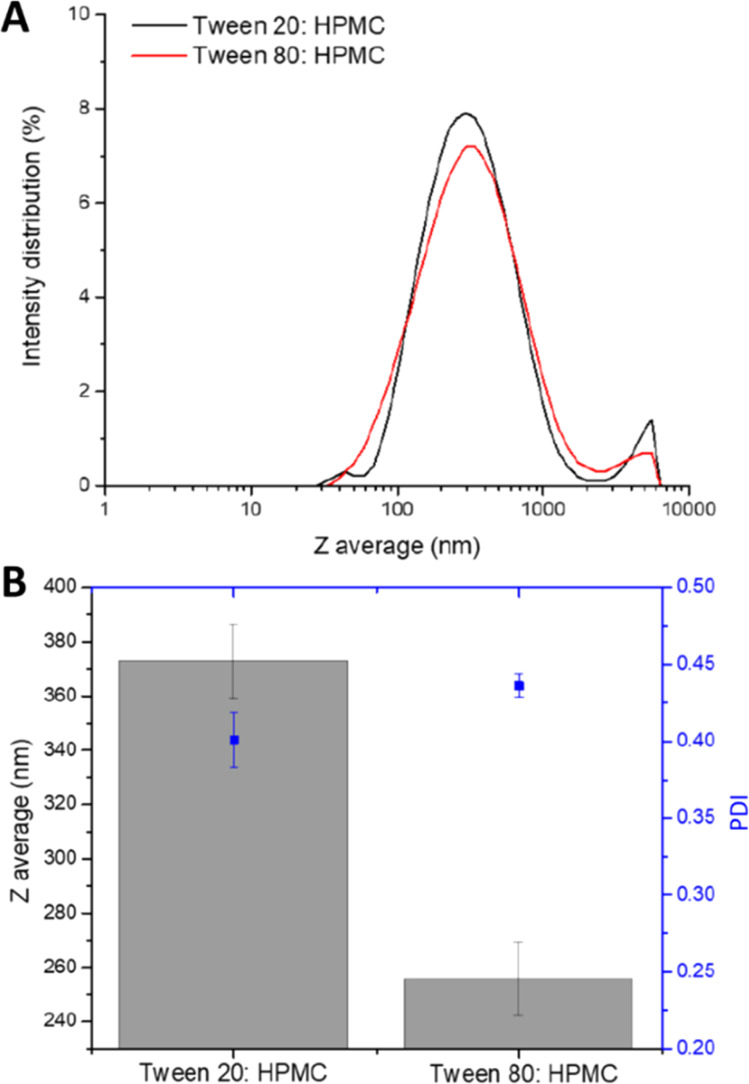
Analysis of the viable indomethacin nanosuspensions producing at 30% wt loading. (A) Size distribution graphs of HPMC:Tween 20 and HPMC:Tween 80 30% wt indomethacin nanosuspension as measured by DLS. (B) Reproducibility of 30% wt indomethacin nanosuspension formulations as measured by DLS to determine the mean diameter and PDI (in blue) using HPMC as the polymeric stabiliser and either Tween 20 or Tween 80.

API loading within any formulation is a key parameter, as it directly controls the API dose administered for a given mass of the total formulation. However, in the context of the ETFD process, higher API loadings typically reduce the number of viable nanosuspension formulations; previous studies have shown that increasing the API content can lead to larger particle sizes that exceed the desired nanoscale range.^15^ Therefore, to identify indomethacin samples with increased API loading, all 42 formulations were produced at 30% wt indomethacin, this was achieved by increasing the concentration of indomethacin dissolved in the organic phase. A 30% wt loading as selected as it offered a three times increase in theoretical API loading compared to the first 10% wt screen but would likely still produce viable formulations. However, the results of the analysis showed that no formulations were found that met the same criteria as applied to the 10% wt formulations. Therefore, the assessment criteria were slightly relaxed to a mean diameter of <600 nm, a standard deviation <10%, and a PDI <0.5. Fig. 2B highlights that with an increased drug loading, there was a significant decrease in the number of viable formulations identified even with the less strict assessment criteria, with only three binary combinations remaining successful. Interestingly, all three viable formulations contained the same polymeric stabiliser, HPMC. Previous research has suggested that HPMC has the ability to maintain its stabilising properties through the freeze drying process of nanoparticles.^36^ The smallest indomethacin nanosuspensions were produced with combinations of HPMC and either Tween 20 or Tween 80 with mean diameters and PDI values of 265 nm, PDI 0.39 (Tween 20) and 247 nm, PDI 0.42 (Tween 80). The DLS intensity size distribution for HPMC: Tween 20 and HPMC: Tween 80 are shown in Fig. 3A. Both the size distributions showed a secondary, larger population in both samples which may be attributed to a low concentration of incompletely dispersed material or aggregated indomethacin nanoparticles. The binary combinations containing HPMC and Tween 20 or Tween 80 were tested for reproducibility across triplicate samples and characterised by DLS (Fig. 3B). Interestingly, triplicate samples containing Tween 80 had a smaller average mean diameters (255 ± 13 nm PDI = 0.4 ± 0.02) compared to those containing Tween 20 (372 ± 14 nm, PDI = 0.4 ± 0.001). This may be attributable to the longer fatty acid chain associated with Tween 80 (C_18,_ oleic acid) *vs.* Tween 20 (C_12,_ lauric acid) potentially leading to stronger hydrophobic association of the surfactant onto the surface of the nanosuspensions. The colloidal stability requirements for any nanoformulation will depend on the method of administration, as nanosuspensions produced by the ETFD process can be stored in the dry form prior to administration long-term stability in the dispersed form is less important than for nanosuspensions stored in the dispersed form. Within this work we are not focussed on a specific route of administration, rather we aim to understand how the formulation composition controls the overall properties of the nanosuspension. In the case the HPMC and either Tween 20 or Tween 80 formulations produced here, they displayed considerable sedimentation 6 hours after redispersion (Fig. S2). In this case, limited colloidal stability of the formulation would require dispersion immediately before administration to avoid changes in particle size distribution potentially altering the bioavailability of the formulation. While it was possible to produce indomethacin nanosuspensions with 30% wt drug loading, the applicability of the formulations would likely be limited due to the short-term colloidal stability.

### Synthesising prodrugs of indomethacin

It was hypothesised that the esterification of the carboxylic acid functionality would increase the viability of the resulting nanosuspension formulations by reducing the solubility of the compound and removing the potential for the ionisation of the carboxylic acid group. These prodrugs have the potential to be activated by naturally occurring carboxylesterase enzymes to activate the prodrug to release the parent indomethacin API. Indeed, this ester cleavage approach has been shown to provide extended release profiles for other APIs.^37–39^

**Fig. 4:**
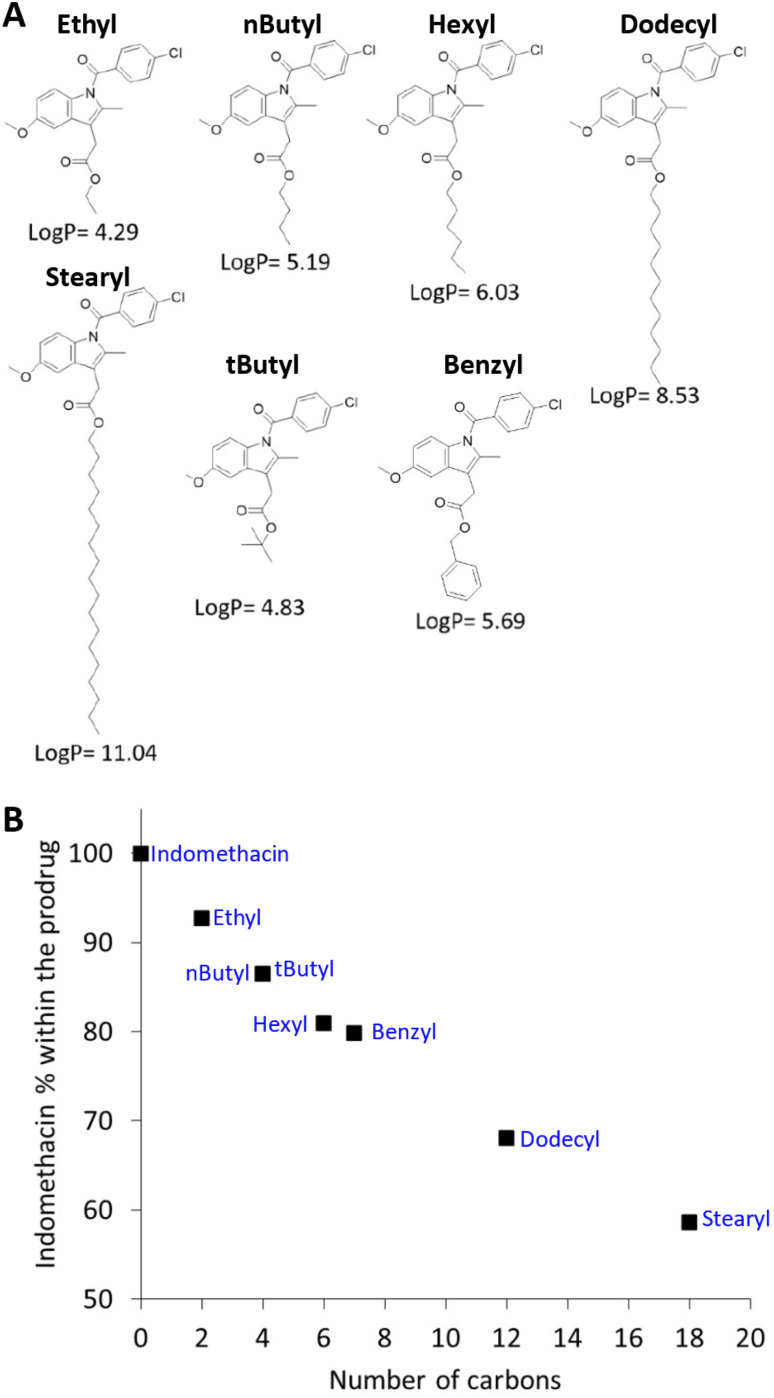
Production of hydrophobic esterified prodrugs of indomethacin. (A) The structures of the hydrophobically modified indomethacin esters synthesised and their log *P* values. (B) The relationship between the mass percentage of the indomethacin API within the prodrug based on the alkyl/aryl modification.

A library of seven hydrophobically modified prodrugs of indomethacin were synthesised by using different alkyl alcohols and one aryl alcohol (Fig. 4A) through the Steglich esterification process.^40^ The seven compounds were designed to enable the investigation of the effect of log *P* and structure of the hydrophobic modification on behaviour of the prodrugs when processed through the ETFD method. Firstly, to synthesise the prodrugs, dicyclohexylcarbodiimide (DCC) was used as a coupling agent and 4-dimethylaminopyridine (DMP) as a catalyst. After synthesis, the crude mixtures were purified through a silica flash column chromatography and characterised by ^1^H and ^13^C nuclear magnetic resonance (NMR), mass spectrometry (MS), elemental analysis and infrared (IR) spectroscopy. The resulting seven hydrophobically modified prodrugs had calculated log *P* (log *P*) values in the range of 4.29–11.04 (Fig. 4A). As with all prodrug strategies, the modification produced compounds with a lower API content than the parent API compound, with higher molecular weight alcohols resulting in lower indomethacin content in the final product (Fig. 4B).

**Fig. 5:**
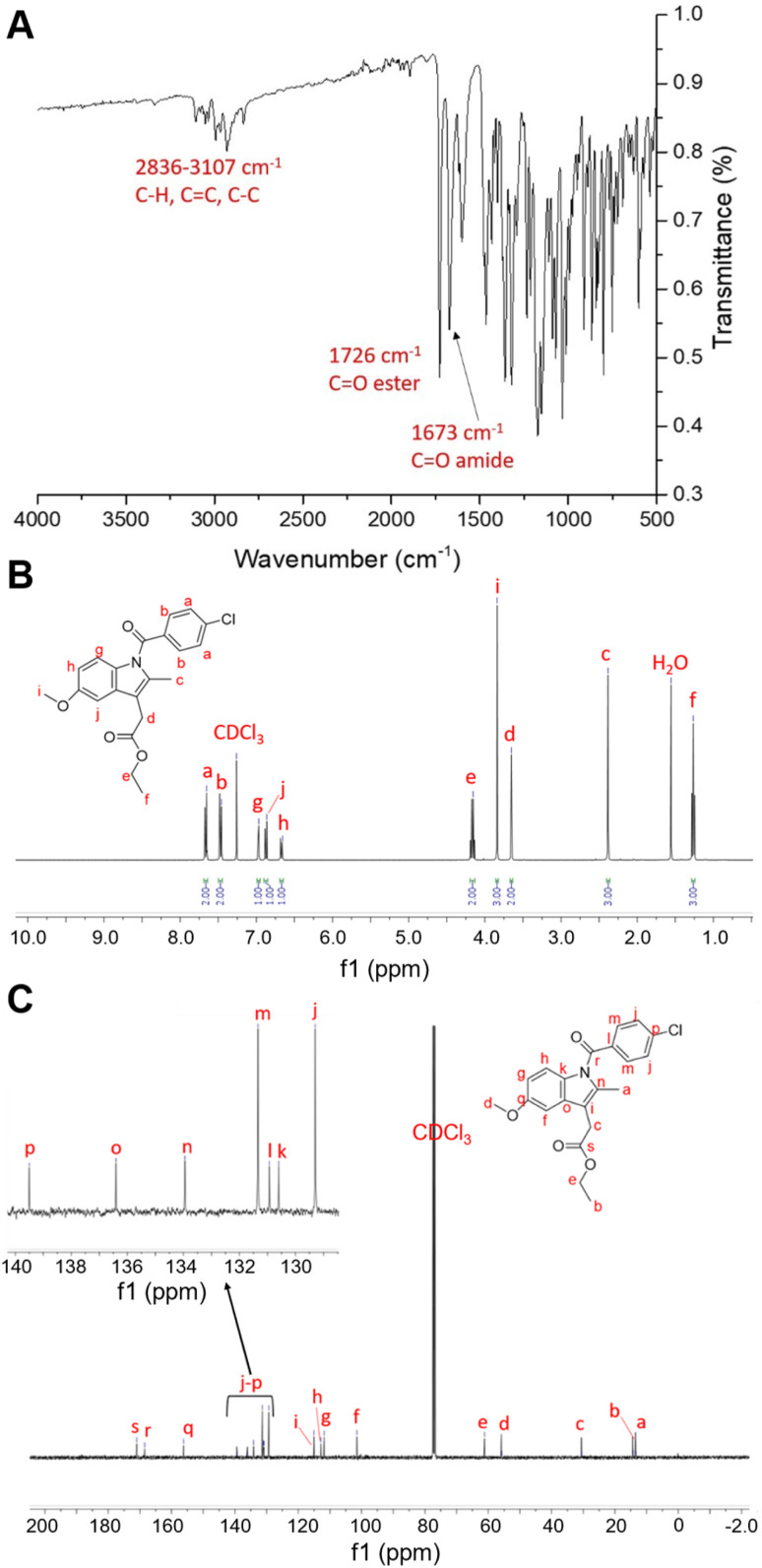
Characterisation of the ethyl ester indomethacin prodrug. (A) FTIR spectra of the indomethacin ethyl ester showing significant carbonyl stretches at 1726 (ester) and 1673 cm^−1^ (amide). (B) ^1^H NMR (CDCl_3_, 400 MHz) spectrum for the indomethacin ethyl ester. (C) ^13^C NMR (CDCl_3_, 400 MHz) spectrum for the indomethacin ethyl ester, the inset is focussed on the region 130–140 ppm.

We exemplify the successful synthesis of the prodrugs with the ethyl prodrug. For the ethyl prodrug, analysis by FTIR showed significant peaks at 1726 cm^−1^ (C=O stretch, ester), 1673 cm^−1^ (C=O stretch, amide), and several peaks between 2836–3107 cm^−1^ (aromatic and non-aromatic C–H stretches) (Fig. 5A). ^1^H NMR analysis showed an increase in the number of hydrogen environments in comparison to indomethacin (see Fig. S3). This was attributed to the successful addition of the ethyl group which gives rise to the quartet at 4.15 ppm and the triplet at 1.26 ppm, peaks correspond to environments e and f, respectively (Fig. 5B). There were 19 ^13^C environments identified as expected shown in Fig. 5C, with additional peaks corresponding to the hydrophobic modification that were not found in indomethacin (C NMR for indomethacin can be seen in Fig. S4). Additionally, elemental analysis of the ethyl indomethacin prodrug and the molecular ion peak identified by mass spectroscopy matched those expected as shown in Table S1. For data on the characterisation of all the other indomethacin prodrugs please see the SI Fig. S5 *n*-butyl, S6, hexyl, S7 dodecyl, S8 stearyl, S9 *t*-butyl and S10 for the benzyl prodrugs.

### Producing nanosuspensions of the indomethacin prodrugs

The seven different indomethacin prodrugs were all processed by the ETFD method targeting a 30% wt loading of the prodrug, *i.e.* each 1 mg of the prodrug nanosuspension formulation will contain a theoretical 0.30 mg or prodrug, 0.47 mg of the polymer stabiliser and 0.23 mg of the surfactant stabiliser. Due to the nature of the modification to produce the prodrugs, the actual loading of indomethacin within the formulation was dependent on the molecular weight of the alkyl or aryl component. For example, a 30 wt% loading of the stearyl ester prodrug (as the highest molecular weight compound) translated to theoretical drug loading of 17.5 wt% of indomethacin. As previously, the different indomethacin prodrugs nanosuspensions were assessed against the viability criteria of a mean diameter and <400 nm and PDI values <0.4, and the number of viable formulations for each hydrophobic prodrug was determined.

**Fig. 6:**
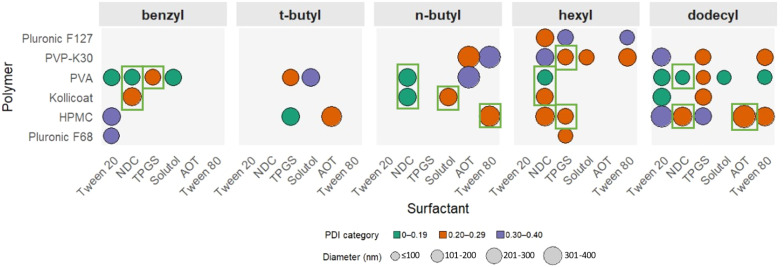
Mean particle properties (mean diameter and polydispersity index as measured by DLS) produced from the ETFD screening of indomethacin ester prodrugs at 30% wt. loading (benzyl, *t*-butyl, *n*-butyl hexyl and dodecyl) with binary combinations of polymer and surfactant stabilisers that produced viable formulations. Note that the ethyl and stearyl prodrugs and the polymer stabiliser PEG 1 K are omitted as they produced no viable formulations. The viable samples are shown by circles representing the successful binary combinations of stabilisers. Green boxes around the samples indicate that the samples met the more restrictive assessment criteria of a mean diameter of <350 ± 50 nm and PDI = ≤0.3 ± 0.1.

Across the different indomethacin prodrugs, a total of 45 viable formulations were identified. The ethyl and stearyl prodrugs did not produce any formulations that were within the viability criteria. A summary of the particle properties of these viable nanosuspension formulations is shown in Fig. 6 (the diameter values can be seen in Table S2), the mean upper diameter of the formulations was determined by the selection criteria (<400 nm) and the largest viable formulations found had a mean diameter of 375 nm. There was no apparent trend across all indomethacin esters with regards to mean particle diameter or PDI. Additionally, there was considerable variability in the combinations of stabilisers that produced viable formulations for the different esters. For example, when the API was the *n*-butyl prodrug there were no viable formulation found for when TPGS was used as a stabiliser, while this same stabiliser produced four viable formulations for both the hexyl and dodecyl esters. One observation was that when a viable formulation was identified for a binary combination containing Kollicoat Protect (7 formulations), there was an 85% chance that the same surfactant would produce a viable formulation with PVA as the polymer stabiliser too. This behaviour may be result of the composition of Kollicoat Protect which is a blend of 55–65% polyvinyl alcohol–polyethylene glycol graft copolymer with 35–35% PVA, suggesting that the PVA within the Kollicoat Protect may be providing a key role in the formation of the viable formulations. Further investigations would be required to understand mechanism of this finding.

By analysing the cumulative frequency of formulations across the different prodrugs it was possible to identify the specific stabilisers and combinations of stabilisers that produced more viable formulations than others (Fig. 8). The most effective stabiliser was PVA which produced 14 viable formulations, while HPMC was the second most effective stabiliser with 12 formulations. The difference in the performance of PVA as a stabiliser for most of the prodrugs compared to unmodified indomethacin may be due to differences in the strengths of the interactions between PVA and the API.^41^ Both PVA and HPMC stabilisers have commonly been reported as stabilisers for colloidal drug delivery systems.^42,43^ For example, the use of HPMC is able to influence the cytotoxicity and release profiles of docetaxel as a chemotherapeutic agent.^44^ PVA has often been reported in many of the reported nanosuspension formulations produced by EFTD.^15,19,20^ With regards to the PVA used in our work, this was a partially hydrolysed PVA (88% hydrolysed) and therefore contains 12% vinyl acetate monomer residues. Both PVA and HPMC are composed on statistically organised hydrophobic and hydrophilic repeat units and as such can adsorb onto particle surfaces by a combination of hydrogen bonding and hydrophobic interactions in an irregular manner along the polymer backbones.^45,46^ Out of the polymer stabilisers tested in our screening experiments, only PVA, Kollicoat Protect and HPMC possessed such statistical organisation of hydrophilic and hydrophobic characteristics (the other polymers were either block copolymers (pluronic F68 an pluronic F127) or uniform in character along the backbone (PVP)), which may suggest that this structure is beneficial for stabilising indomethacin-derived nanosuspensions produced by ETFD.

In terms of the surfactant stabilisers, TPGS and NDC were found to be the most effective (11 formulations contained these stabilisers). These two stabilisers possessed rather different properties; NDC is negatively charged and therefore provides electrostatic repulsion between the particles in suspension. As a small molecule stabiliser, it has the potential for rapid diffusion to the particle surface to provide enhanced stability.^44^ TPGS however, is a non-ionic amphiphilic surfactant and therefore provides steric stabilisation through the adsorption of the lipophilic tocopheryl group. As such, it is not possible to identify commonalities between these two successful stabilisers for the APIs tested. Interestingly, all the stabilisers, except PEG, produced at least one viable formulation in the different combinations tested. Notably PEG and PVP were the only polymer stabilisers used in the screening that did not possess any amphiphilic character variation along their polymer chains. However, the use of PVP as the stabiliser resulted in 7 viable formulations. Indeed, PVP has been widely used as a colloidal stabiliser as it possesses amphiphilic character within each repeat unit due to the hydrophobic alkyl backbone and hydrophilic pyrrolidone rings as the side groups.^13^

**Fig. 7:**
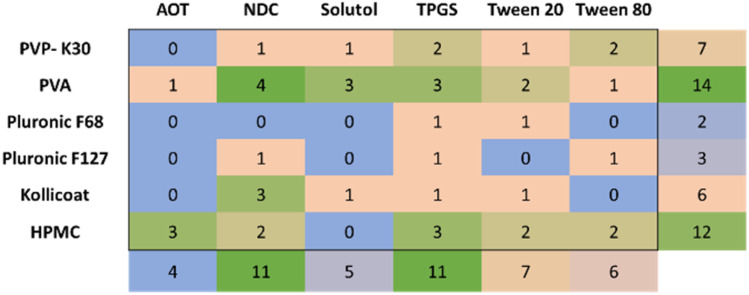
A summary of the number of viable formulations produced for the different stabiliser combinations across all the indomethacin prodrugs at 30% wt. loading.

Furthermore, some formulations seemed to display a synergy between stabilisers, for example, dioxtyl sulfosuccinate sodium salt (AOT) only produced viable formulations for two stabiliser combinations (either with HPMC or PVA), however, the AOT-HPMC combination produced a total of three viable formulations which was the, only slightly less than the most successful formulation; NDC-PVA combination which produced four viable formulations (see Fig. 7). This data shows how the APIs and stabilisers that make up formulations appear to have synergies that determine the success in producing a viable formulation. These interactions make it hard to predict viable formulation even for any API with systematic physiochemical changes.

**Fig. 8:**
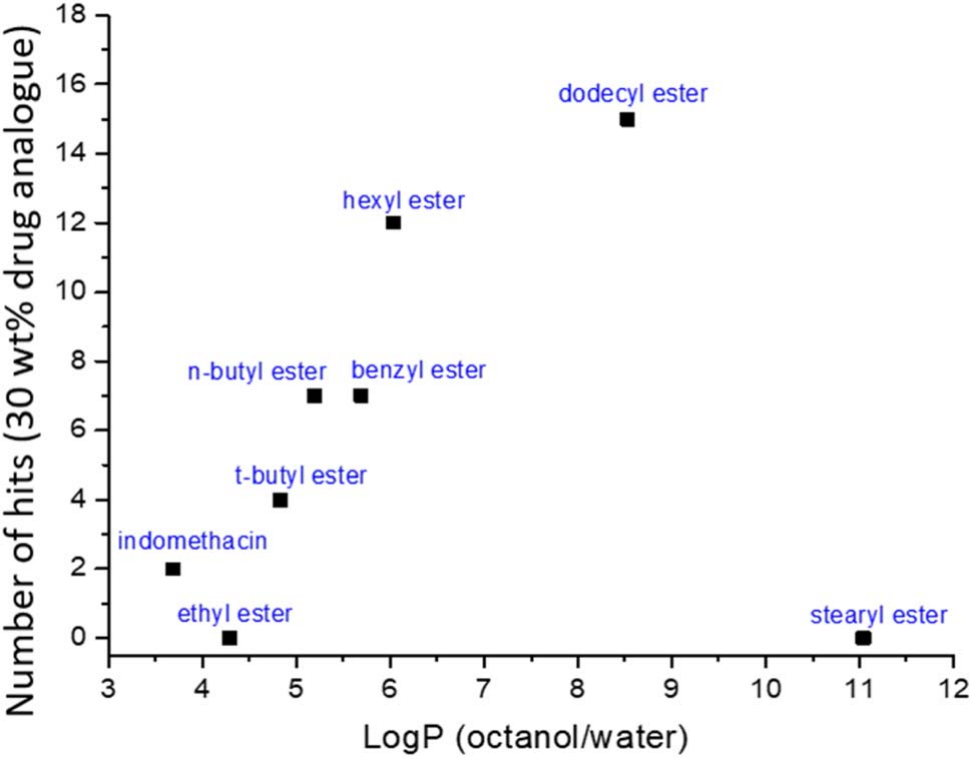
The relationship between the number of viable samples achieved for each indomethacin prodrugs at 30 wt% loading and the calculated log *P* of the prodrug.

By considering the number of viable formulations and the log *P* of the prodrug (with the exception of the stearyl prodrug which did not produce any viable formulations) a positive relationship was observed where generally prodrugs with higher log *P* resulted in more viable formulations (see Fig. 8). This relationship is hypothesised to be because higher log *P* values indicate molecules with increasing hydrophobicity, the consequent increase in hydrophobic character, might increase hydrophobic interactions between the prodrugs alkyl chains and the stabiliser. In prior work, Tóth *et al.* have shown similar trend using 4-hydroxy benzoate as a model drug which was esterified with increasing alkyl chain lengths, the associated increase in log *P* was found to correlate with an increased encapsulation efficiency of their target molecules within polymer nanoparticles.^47^ Additionally, a higher log *P* of an API has also been shown to increase the supersaturation conditions required for nanoparticle nucleation in the aqueous phase, thus further increasing the success of nanoparticle formation.^48^ However, the most hydrophobic prodrug, the stearyl ester (log *P* = 11.04) failed form any viable nanosuspension formulations when processed by ETFD. Longer alkyl chains have been shown to increase the crystallization tendency of another API (paliperidone),^49^ and long alkyl groups have been shown to aggregate through a process known as “nanophase separation”.^50^ Therefore, for our formulation it may be that the increased interaction between the prodrug molecules themselves may might limit the ability for the stabilisers–prodrug interactions. To further understand this behaviour future work should look at the impact of longer alkyl chain modifications.

### Assessing the stability of the formulations

To further refine the formulations to obtain formulations with more narrowly defined particle size distributions, a stricter set of criteria. Here, a lower mean diameter (<350 ± 50 nm) and a reduced PDI ≤0.3 ± 0.1) were applied to the 45 viable samples to give 13 formulations (see Fig. 6, green boxes). These parameters were selected because smaller particles possess faster dissolution behaviour and narrower size distributions are known to slow Ostwald ripening.^51^ Ostwald ripening is a process by which larger particles grow as the expense of smaller particle due to transport of soluble API through the continuous phase is a common cause of stability issues in colloidal systems such as nanosuspensions.^52,53^ The DLS mean diameter and PDI data for these 13 formulations can be found in Fig. S11A, showing that all these formulations were reproducible with a variation of ∼10% of the diameter. Out of these 13 formulations, the NDC-PVA stabiliser combination was found to work for four different prodrugs, those with benzyl, *n*-butyl, hexyl and dodecyl modification. Selection of these samples allowed for different the behaviour prodrugs to be compared with the same pair of stabilisers. DLS analysis of these formulations showed narrow, predominately monomodal, size distributions (Fig. S11B) with the mean diameters of the formulations all in the range 160–240 nm. The theoretical indomethacin loadings of these dry formulations were 26%, 24%, 24% and 20% for the *n*-butyl, benzyl hexyl and dodecyl prodrug formulations respectively.

**Fig. 9:**
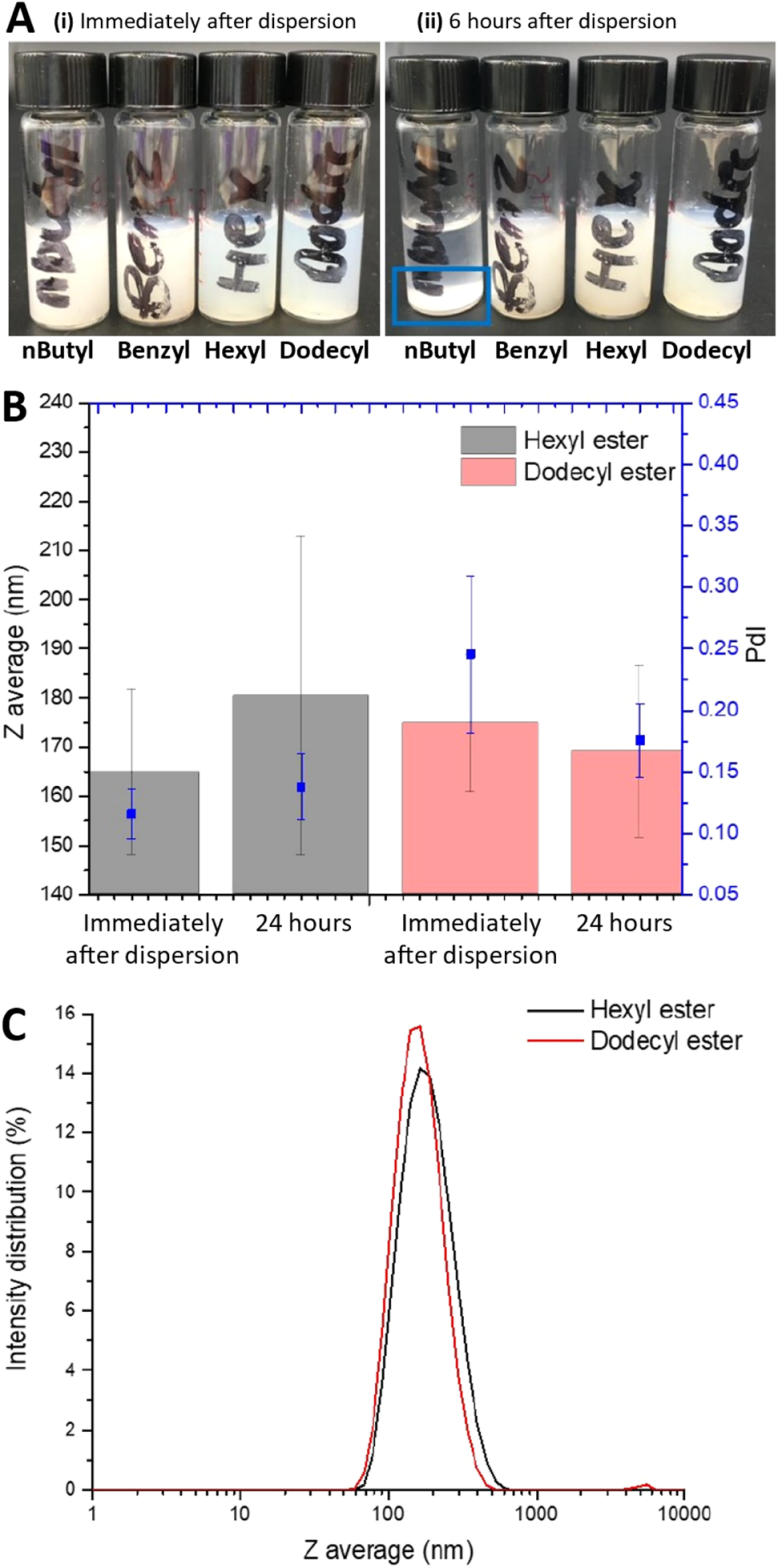
Stability and particle properties of the 30 wt% indomethacin prodrug nanosuspension samples with NDC-PVA as the stabiliser combination. (A) Photo of the after (i) immediate dispersion when redispersed at 1 mg mL^−1^ active in PBS (0.01 M) and (ii) after 6 hours of dispersion. The blue box highlights the sedimented solid seen with 30 wt% *n*-butyl indomethacin analogies. (B) Comparison of the mean diameter of the hexyl prodrug and dodecyl prodrug before and after 24 hours in the dispersed form. (C) DLS size intensity distributions of the hexyl ester and the dodecyl ester prodrugs 24 hours after dispersion.

These four formulations were selected to assess how the different modifications of the indomethacin prodrugs influenced the dispersion stability of the formulations. To ensure that the formulation displayed sufficient dispersion stability for preparation and dosing, the freeze-dried monoliths were dispersed and left for six hours to allow comparison with the stability shown for the indomethacin (non-prodrug) which showed extensive sedimentation after this duration (Fig. S2). Immediately after dispersion the formulations formed white turbid, homogeneous dispersions, with all formulations displaying similar turbidity (Fig. 9A(i)). However, six hours after dispersion, sedimentation of *n*-butyl formulation had occurred (Fig. 9A(ii)) but not for the other three prodrug formulations. After 24 hours, the benzyl prodrug had also sedimented, while the hexyl and dodecyl prodrugs did not display any visually detectable sedimentation. DLS analysis of these two formulations showed negligible change mean diameter or PDI before or after 24 hours in the dispersed form (Fig. 9B). Additionally, both the hexyl and dodecyl prodrugs formulations maintained uniformity within the dispersions as emphasised by narrow PdI values (≤0.25) and monomodal size distributions as shown in Fig. 9C. Given that all four of these formulations possessed similar initial mean diameters, the tendency of the formulations of *n*-butyl and benzyl prodrugs to sediment indicated that particle growth was occurring in the formulations that sedimented; larger particles sediment at a faster rate than smaller particles. Unfortunately, it was not possible to obtain accurate DLS measurements on the *n*-butyl and benzyl prodrugs formulation due to the presence of large particles that were above the size range acceptable for DLS analysis. Reducing the solubility of the API or adding in a small amount of very poorly soluble hydrophobe has been shown to inhibit Ostwald ripening.^52^ While the log *P* value of a compound does not directly correlate the compound's water solubility, generally the log *P* of a compound is inversely proportional to aqueous solubility.^54^ In the case of our formulations, it is likely that the lower solubility of these hexyl and dodecyl prodrugs greatly slowed Ostwald ripening, resulting in increased stability of the formulation of the prodrugs. Conversely, the *n*-butyl and benzyl prodrugs with lower log *P* values (and likely higher aqueous solubilities) would have experienced faster Ostwald ripening driving particle growth and ultimately sedimentation. Furthermore, as previously mentioned, all nanosuspension formulations of the indomethacin parent compound also showed considerable sedimentation 6 hours after redispersion. The requirements for specific durations of dispersion stability for any nanomedicine formulation depends on the how the sample is stored, prepared and the route of administration. In the case of a freeze-dried formulation for potential oral dosing, such a sample has the potential to be dispersed immediately prior to oral ingestion or administered in the drug form to be dispersed upon. Alternatively, ocular administration (where it is used to manage ocular inflammation) the nanosuspension can be dispersed prior to administration. As such, we believe that the 24 hours of colloidal stability in the dispersed form may be sufficient for such applications. Ultimately, our stability analysis showed the importance of hydrophobic modification; the two indomethacin prodrugs with the highest log *P* values displayed considerably improved dispersion stability compared to less hydrophobic prodrugs.

An additional benefit of the ETFD process is that it yields freeze dried monoliths with potential for long term storage in the dried form prior to use. Therefore, the hexyl and dodecyl ester indomethacin prodrug monoliths were assessed in terms of their storage stability of at room temperature after freeze drying. The samples were analysed in triplicate after six weeks storage and immediately assessed by DLS in the dispersed form for 24 hours. Upon dispersion, the hexyl ester prodrug had a mean diameter of 180 nm which gradually increased over 24 hours to have a mean diameter of 250 nm (Fig. S12). These mean particle diameters were within the range of values we measured prior to storage (Fig. 9B). Similar trends in the particle diameter growth have been reported for other nanosuspensions, where a lopinavir nanosuspensions displayed an increase in mean diameter of 40% (500 to 700 nm) and decrease in PDI from 0.4 to 0.25 over a 10 hours period.^19^ Conversely, after six weeks storage DLS analysis of the dodecyl formulation revealed a sample unsuitable for DLS analysis due to the presence of larger sedimenting particles, preventing accurate DLS diameter measurements from being obtained. These findings suggest that the dodecyl indomethacin prodrug monolith was subject to destabilisation upon storage. The reason for this behaviour is not fully understood and will require further investigated in the future. Our assessment of storage stability shows that relatively small changes in the chemical structure can affect the storage stability of nanosuspensions produced by EFTD.

## Conclusions

This study presents the first systematic structure-formulation investigation of indomethacin ester prodrugs for the production of nanosuspensions *via* emulsion-templated freeze drying (ETFD). By synthesising a panel of seven prodrugs with varying alkyl and aryl ester groups, we demonstrate that even subtle changes in prodrug structure can significantly influence formulation success. Our results show that intermediate hydrophobicity favours the formation of well-dispersed, submicron particles across a broader range of stabiliser combinations. Importantly, we identify consistent trends in stabiliser performance, with selected binary combinations enabling robust formulation of multiple prodrugs.

These insights offer practical guidance for rational prodrug design in nanosuspension development, enabling more efficient formulation screening. In doing so, this work contributes to a deeper understanding of the interplay between API structure and colloidal behaviour during ETFD processing. The findings provide a foundation for expanding nanosuspension formulation beyond empirical screening, which may accelerate the development of new long-acting and poorly soluble drug candidates using scalable, solid-state nanomedicine platforms.

## Materials and methods

### Materials

Indomethacin (≥99%), poly(ethylene glycol) methyl ether (average M_*n*_ 1000), Pluronic^®^ F68, Pluronic^®^ F127, polyvinyl alcohol (PVA) (grade 4–88, MW 57–77,000 g mol^−1^), hydroxypropyl methylcellulose (HPMC) MW = 10 000 g mol^−1^(1.8–2.0 mol methoxy per mol cellulose, 29 wt% methoxy, 0.2–0.3 mol propylene oxide per mol cellulose, 7 wt% propylene oxide), polyvinyl alcohol–copolyethylene glycol (Kollicoat protect), polyvinyl pyrollidone K30 (PVP-K30), tween 20, tween 80, analytical grade ethanol, *n*-butanol, *n*-hexanol, *n*-dodecanol, *tert*-butanol, benzyl alcohol, d-α tocopheryl polyethylene glycol 100 succinate (TPGS), sodium deoxycholate (NDC), dioxtyl sulfosuccinate sodium salt (AOT), polyethylene glycol (15)-hydroxy stearate (Solutol), dicyclohexylcarbodiimide (DCC), anhydrous dichloromethane (DCM) and 4-dimethylamino pyridine (DMAP) were all purchased from Sigma Aldrich. 1-Propanol (HPLC grade), 2-propanol (IPA, HPLC grade), acetonitrile (HPLC grade), ethyl acetate, *n*-hexane and analytical grade acetone were purchased from Fisher Scientific. All materials were used as received.

### General method for emulsion templated freeze drying (ETFD)

Polymer and surfactants were weighed out into separate 14 mL glass sample vials and dissolved to a final concentration of 22.5 mg mL^−1^ in distilled water. These solutions were left overnight on a rolling mixer to ensure thorough dissolution. The API used (indomethacin or an indomethacin prodrug) was dissolved at 10 mg mL^−1^ in chloroform to achieve a 10 wt% drug loading in the final formulation (for indomethacin or the indomethacin prodrugs), or 30 mg mL^−1^ for the 30 wt% formulations of the indomethacin prodrugs. In all cases, the solution was left on a rolling mixer for 1 hour to ensure thorough dissolution. For the preparation of each nanosuspension sample, 103 μL of surfactant solution, 207 μL of polymer solution, 90 μl of water and 100 μL of prodrug solution were added to a 4 mL glass sample vial. This was repeated for all 42 combinations of polymer and surfactant (7 × 6) and for each API compound. To produce an emulsion the 1 : 4 ratio of chloroform to aqueous phase was sonicated for 30 s with the following protocol: 20% duty cycle; 250 intensity; 500 cycles/burst; frequency sweeping mode (giving an average output of 70 W). Samples were sonicated in a temperature-controlled water bath set to 4 °C. Immediately after sonication, emulsion samples were frozen in liquid nitrogen, prior to freeze drying using a VirTis BenchTop K freeze dryer (SP Scientific, Ipswich, UK) with condenser temperature set to −100 °C and vacuum of <40 μbar. Sample remained in the freezer dryer for 48 hours, after which they were sealed air-tight and stored in a desiccator at ambient temperature, prior to analysis. The final composition yielded a freeze dried monolith containing 3 mg indomethacin ester (30 wt%), 2.3 mg surfactant (23 wt%), and 4.7 mg polymer (47 wt%).

### Generic method for the esterification of indomethacin to produce the indomethacin prodrugs

Indomethacin (3 g, 0.008 mol, 1 eq.) was dissolved in the minimum amount of anhydrous DCM required (∼60 mL) to form a bright yellow solution that was degassed with N_2_ for 10 minutes. DMAP (0.15 g, 0.0167 mol, 0.15 eq.) and the chosen alcohol for esterification (2 eq.) was dissolved in anhydrous DCM. The DMAP/alcohol for esterification were added under N_2_ to the IND/DCM mixture. Following this, DCC (2.59 g, 0.0126 mol, 1.5 eq.) was dissolved in DCM (20 mL) and added slowly to the mixture at 0 °C and under N_2_ whilst stirring. Upon the addition of DCC the solution turned from transparent yellow to a cloudy suspension. Following the complete addition of DCC, the reaction mixture was warmed to room temperature and left for 48–72 hours. The completion of the reaction was determined through TLC. After reaction completion white precipitate of the side product DCU was filtered by gravity. The solvent from the resultant filtrate was removed *in vaccuo* and the crude solid was re-suspended in the minimum amount of cold EtoAc. Residual DCU precipitated and the reaction was filtered by gravity again. The crude product was washed with NaHSO_4_ (2 × 50 mL) to remove excess DMAP, Na_2_CO_3_ (2 × 50 mL) to remove unreacted drug, followed by DI water (1 × 100 mL) and brine (1 × 100 mL). The crude product was then loaded onto silica before purification by flash chromatography using EtOAc: hexane binary eluent systems. The solvent system used was dependent on the different esters synthesised.

The log *P* values for the indomethacin prodrugs were estimated using ChemDraw 18.0 using the “log *P*” value from the “Chemical Properties” function.

### Generic method for the purification of indomethacin esters using column purification and TLC

Thin layer chromatography was performed using Merck Kieselgel 60 F254 aluminium backed silica plates. Visualization was achieved by UV fluorescence or a basic KMnO_4_ solution and heat. Flash column chromatography (FCC) was performed using silica gel (Aldrich 40–63 μm, 230–400 mesh). The crude material was pre-adsorbed onto silica prior to application to the column. All purified products were eluted and analysed by ^1^H NMR, ^13^C NMR, IR, elemental analysis, mass spectrometry and DSC.

#### Ethyl ester

Ethanol (0.77 g, 0.0167 mol, 2 eq.) was used. The final product was presented as an off white solid (1.91 g, 59% yield). TLC analysis in 20: 80 EtOAc: hexane obtained an RF value of 0.3.

^1^H-NMR (400 MHz, CDCl_3_): *δ* ppm = 1.26 (T, 3H), 2.38 (S, 3H), 3.65 (S, 2H), 3.84 (S, 3H), 4.15 (Q, 2H), 6.65 (D of D, 1H), 6.86 (D. 1H), 6.97 (D, 1H), 7.46 (D, 2H), 7.65 (D, 2H). ^13^C NMR (100 MHz, CDCl_3_): all ppm shifts correspond to 1 carbon environment unless otherwise stated: *δ* ppm = 13.40, 14.26, 30.49, 55.71, 61.17, 101.48, 111.81, 112.86, 115.09, 129.26 (2C), 130.85, 130.97, 131.33 (2C), 134.10, 136.03, 139.37, 159.19, 168.46, 171.02. IR (cm^−1^) = 1673 (C=O, amide), 1725 (C=O ester), 2836–3107 (C–H, C=C, C–C). ESI-MS [M + Na]^+^ = 408.1 *m*/*z*. Elemental analysis = calculated: C (65.38), H (5.25), N (3.63), obtained: C (65.37), H (5.22), N (3.53).

#### *n*-Butyl ester

*n*-Butyl alcohol (1.24 g, 0.0167 mol, 2 eq.) was used. The final product was presented as an off white solid (2.58 g, 74% yield). TLC analysis in 20: 80 EtOAc: hexane obtained an RF value of 0.4. ^1^HNMR (400 MHz, CDCl_3_): *δ* ppm = 0.90 (T, 3H), 1.33 (M, 2H), 1.61 (M, 2H), 2.38 (S, 3H), 3.65 (S, 2H), 3.84 (S, 3H), 4.10 (T, 2H), 6.65 (D of D, 1H), 6.86 (D, 1H), 6.97 (D, 1H), 7.46 (D 2H), 7.65 D, 2H). ^13^C NMR (100 MHz, CDCl_3_): all ppm shifts correspond to 1 carbon environment unless otherwise stated: *δ* ppm = 13.49, 13.80, 19.25, 30.57, 30.77, 55.83, 65.06, 101.43, 111.84, 112.90, 115.08, 129.25 (2C), 130.83, 130.95, 131.31 (2C), 134.10, 136.04, 139.8, 159.19, 168.44, 171.07. IR (cm^−1^) = 1668 (C=O, amide), 1724 (C=O, ester), 2386–3003 (C–H, C=C, C–C). ESI-MS [M + Na]^+^ = 413.1 *m*/*z*. Elemental analysis = calculated: C (66.74), H (5.84), N (3.38), obtained: C (66.95), H (5.88), N (3.38).

#### *n*-Hexyl ester

Hexyl alcohol (1.72 g, 0.0167 mol, 2 eq.) was used. The final product was presented as a pale yellow solid (2.81 g, 76% yield). TLC analysis in 20: 80 EtOAc: hexane obtained an RF value of 0.2. ^1^HNMR (400 MHz, CDCl_3_): *δ* ppm = 0.86 (T, 3H), 1.26 (M, 6H), 1.61 (M, 2H), 2.39 (S, 3H), 3.65 (S, 2H), 3.83 (S, 3H), 4.09 (T, 2H), 6.65 (D of D, 1H), 6.86 (D, 1H), 6.97 (D, 1H), 7.46 (D, 2H), 7.65 (D, 2H). ^13^C NMR (100 MHz, CDCl_3_): all ppm shifts correspond to 1 carbon environment unless otherwise stated: *δ* ppm = 13.48, 14.09, 22.63, 25.67, 28.70, 30.58, 31.50, 55.81, 65.32, 101.45, 111.78, 112.91, 115.06, 129.24 (2C), 130.82, 130.94, 131.30 (2C), 134.09, 135.99, 139.36, 156.17, 168.42, 171.09. IR (cm^−1^) = 1689 (C=O, amide), 1722 (C=O, ester), 2834–3093 (C–H, C=C, C–C). ESI-MS [M + Na]^+^ = 441.2 *m*/*z*. Elemental analysis = calculated: C (67.94), H (6.39), N (3.17), obtained: C (68.29), H (6.50), N (3.16).

#### *n*-Dodecyl ester

Dodecanol (3.13 g, 0.0167 mol, 2 eq.) was used. The final product was presented as a viscous yellow oil (2.73 g, 65% yield). TLC analysis in 10: 90 EtOAc: hexane obtained an RF value of 0.4. ^1^H-NMR (400 MHz, CDCl_3_): *δ* ppm = 0.88 (T, 3H), 1.25 (M, 18H), 1.59 (M, 2H), 2.39 (S, 3H), 3.65 (S, 2H), 3.83 (S, 3H), 4.09 (T, 2H), 6.65 (D of D, 1H), 6.86 (D, 1H), 6.97 (D, 1H), 7.46 (D, 2H), 7.65 (D, 2H). ^13^C NMR (100 MHz, CDCl_3_): all ppm shifts correspond to 1 carbon environment unless otherwise stated: *δ* ppm = 13.49, 14.26, 22.83, 26.04, 28.76, 29.37, 29.49, 29.66, 29.71, 29.76, 29.79, 30.59, 32.05, 55.82, 65.35, 101.45, 111.81, 112.91, 115.07, 129.25 (2C), 130.83, 130.95, 131.32 (2C), 134.09, 136.00, 139.38, 156.18, 168.43, 171.10. IR (cm^−1^) = 1668 (C=O, amide), 1726 (C=O, ester), 2838–3005 (C–H, C=C, C–C). ESI-MS [M + Na]^+^ = 548.3 *m*/*z*. Elemental analysis = calculated: C (70.77), H (7.66), N (2.66), obtained: C (70.95), H (7.68), N (2.69).

#### *n*-Stearyl ester

Stearyl alcohol (4.54 g, 0.0167 mol, 2 eq.) was used. The final product was presented as a pale yellow solid (4.24 g, 83% yield). TLC analysis in 30: 70 EtOAc: hexane obtained an RF value of 0.7. ^1^HNMR (400 MHz, CDCl_3_): *δ* ppm = 0.88 (T, 3H), 1.25 (M, 30H), 1.61 (Q, 2H), 2.39 (S, 3H), 3.65 (S, 2H), 3.83 (S, 3H), 4.09 (T, 2H), 6.65 (D of D, 1H), 6.86 (D, 1H), 6.69 (D, 1H), 7.46 (D, 2H), 7.65 (D, 2H). ^13^C NMR (100 MHz, CDCl_3_): all ppm shifts correspond to 1 carbon environment unless otherwise stated: *δ* ppm = 13.82, 14.59, 23.16, 26.36, 29.09, 29.70, 29.83, 30.00, 30.05, 30.13, 30.14 (5C), 30.17, 30.92, 32.39, 56.15, 65.67, 101.78, 112.13, 113.24, 115.40, 129.58 (2C), 131.16, 131.28, 131.64 (2C), 134.42, 136.33, 139.71, 156.51, 168.75, 171.42. IR (cm^−1^) = 1673 (C=O, amide), 1736 (C=O, ester), 2848–2956 (C–H, C=C, C–C). ESI-MS [M + Na]^+^ = 632.3 *m*/*z*. Elemental analysis = calculated: C (72.82), H (8.59), N (2.30), obtained: C (72.69), H (8.66), N (2.32).

#### *t*-Butyl ester

*t*-Butyl alcohol (1.24 g, 0.0167 mol, 2eq.) was used. The final product was presented as an off white solid (2.20 g, 63% yield). TLC analysis in 20: 80 EtOAc: hexane obtained an RF value of 0.5. ^1^H-NMR (400 MHz, CDCl_3_): *δ* ppm = 1.45 (S, 9H), 2.37 (S, 3H), 3.56 (S, 2H), 3.84 (S, 3H), 6.65 (D of D, 1H), 6.87 (D, 1H), 6.96 (D, 1H), 7.46 D, 2H), 7.65 (D, 2H). ^13^C NMR (100 MHz, CDCl_3_): all ppm shifts correspond to 1 carbon environment unless otherwise stated: *δ* ppm = 13.54, 28.24 (3C), 31.88, 55.85, 81.29, 101.51, 111.77, 113.50, 115.07, 129.24 (2C), 130.96, 131.00, 131.30 (2C), 134.19, 135.85, 139.32, 156.15, 168.47, 170.31. IR (cm^−1^) = 1685 (C=O, amide), 1732 (C=O, ester), 2840–3007 (C–H, C=C, C–C). ESI-MS [M + Na]^+^ = 436.1 *m*/*z*. Elemental analysis = calculated: C (66.74), H (5.84), N (3.38), obtained: C (66.73), H (5.86), N (3.40).

#### Benzyl ester

Benzyl alcohol (1.82 g, 0.0167 mol, 2 eq.) was used. The final product was presented as an off white solid (2.68 g, 74% yield). TLC analysis in 20: 80 EtOAc: hexane obtained an RF value of 0.3. ^1^HNMR (400 MHz, CDCl_3_): *δ* ppm = 2.36 (S, 3H), 3.71 (S, 2H), 3.76 (S, 3H), 5.14 (S, 2H), 6.65 (D of D, 1H), 6.87 (D, 1H), 6.93 (D, 1H), 7.32 (M, 5H), 7.45 (D, 2H), 7.63 (D, 2H). ^13^C NMR (100 MHz, CDCl_3_): all ppm shifts correspond to 1 carbon environment unless otherwise stated: *δ* ppm = 13.35, 30.58, 55.78, 66.94, 101.33, 112.02, 112.64, 115.11, 128.31 (2C), 128.45, 128.70 (2C), 129.27 (2C), 130.72, 130.95, 131.32 (2C), 134.05, 135.90, 136.08, 139.41, 156.20, 168.44, 170.80. IR (cm^−1^) = 1660 (C=O, amide), 1720 (C=O, ester), 2846–3113 (C–H, C=C, C–C). ESI-MS [M + Na]^+^ = 470.1 *m*/*z*. Elemental analysis = calculated: C (69.72), H (4.95), N (3.13), obtained: C (69.81), H (4.92), N (3.12).

### Dynamic light scattering

Dynamic light scattering (DLS) were carried out at 25 °C using a Malvern Zetasizer Nano ZS instrument at a nanoparticle concentration of 1 mg mL^−1^. All measurements were taken using standard conditions at 25 °C: a laser wavelength of 630 nm, a fixed backscattering angle of 173° using automated setting for measurement position selection and attenuator selection. The dispersed phase viscosity was 1.33 m.Pa.s. All measurements of individual samples were taken in triplicate and the Z-average and PDI taken from the average of the triplicate measurements.

### Nuclear magnetic resonance

H and ^13^C nuclear magnetic resonance (NMR) spectra were recorded in CDCl_3_ using a Bruker Avance spectrometer operating at 400 and 100 MHz respectively. Chemical shifts (*δ*) are reported in parts per million (ppm) and TMS was used as an internal standard for both ^1^H and ^13^C NMR spectra.

### Electrospray mass spectrometry

Electrospray (ESI) mass spectrometry data were recorded in the Mass Spectrometry Laboratory at the University of Liverpool using a MicroMass LCT mass spectrometer using electron ionisation and direct infusion syringe pump sampling. All materials were diluted with methanol. Dilution concentration was dependent on the molecular weight of the entity.

### Elemental analysis

Elemental analyses were obtained from a Thermo FlashEA 1112 series CHNSO elemental analyser.

## Conflicts of interest

The authors declare that they have no conflict of interest.

## Supplementary Material

RA-015-D5RA06900A-s001

## Data Availability

The data supporting this article have been included as part of the supplementary information (SI). Supplementary information: detailed characterisation data for all synthesised indomethacin ester prodrugs, including ^1^H and ^13^NMR and FTIR spectra, along with elemental and mass spectrometry data; the structures of all stabilisers used in the formulation screening, full nanosuspension screening datasets (particle diameter and PDI for each formulation), and reproducibility and stability studies. Additional DLS data demonstrate the temporal dispersion stability of selected nanosuspensions and support the reproducibility of the ETFD process. See DOI: https://doi.org/10.1039/d5ra06900a.
