# Supplementary material for: The synthesis of indomethacin prodrugs for the formation of nanosuspensions by emulsion templated freeze drying
Source: RSC Adv. 2025 Nov 11;15(51):43830–43. doi: 10.1039/d5ra06900a (PMC12603901; doi:10.1039/d5ra06900a)
Supplement: RA-015-D5RA06900A-s001 [file RA-015-D5RA06900A-s001.pdf]

Supplementary Information (SI) for RSC Advances.
This journal is © The Royal Society of Chemistry 2025

The Synthesis of Indomethacin Prodrugs for the Formation of Nanosuspensions by Emulsion Templated Freeze Drying

Jessica Taylor, Andrew Sharp, Steve P. Rannard, Sarah Arrowsmith and Tom O. McDonald

Supporting Information

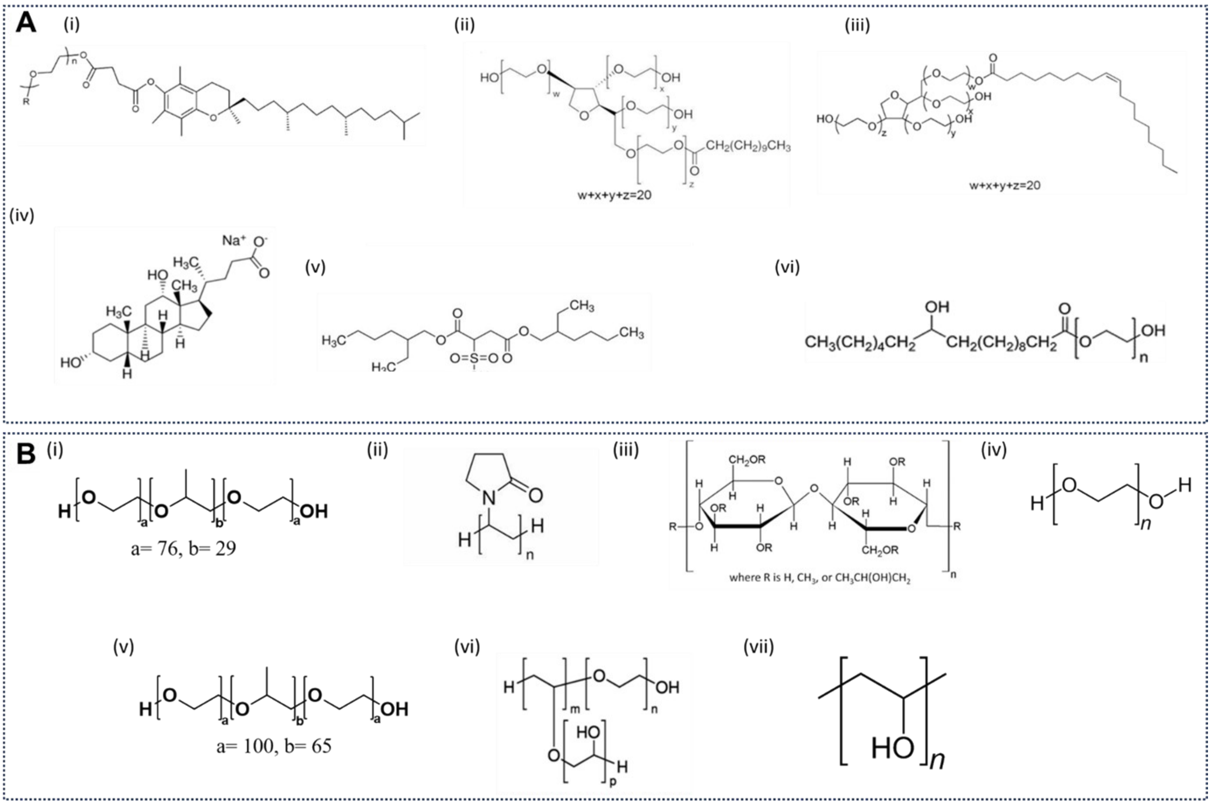

Figure S1: Structures of the six surfactants and seven polymeric stabilisers employed in the screening process of nanosuspensions formulations. A) Represents the surfactants used, all denotations for the individual excipients are shown in brackets after each surfactant: (i) d-α-tocopheryl polyethylene glycol 1000 succinate (TPGS) (ii) Tween 20, (iii) Tween 80, (iv) Sodium deoxycholate (NDC) (v) Dioxytl sulfosuccinate sodium salt (AOT) and (vi) Polyethylene glycol (15)-hydroxyl stearate (Solutol). B) Represents the polymeric stabilisers (i) Polyethylene glycol$_{76}$-polypropylene glycol$_{29}$-polyethylene glycol$_{76}$ (Pluronic® F68), (ii) Polyvinylpyrrolidone K30 (PVPK30) (iii) Hydroxypropyl methylcellulose (HPMC), (iv) Polyethylene glycol 1000 (PEG 1K) (v) Polyethylene glycol$_{100}$-polypropylene glycol$_{65}$-polyethylene glycol$_{100}$ (Pluronic® F127),(vi) Polyvinyl alcohol-polyethylene glycol copolymer and polyvinyl alcohol (Kollicoat Protect) and (vii) Polyvinyl alcohol (PVA). Note that Kollicoat Protect is a multicomponent product it is a composition of 55-65% polyvinyl alcohol-polyethylene glycol graft copolymer (brand name Kollicoat IR), 35-45% PVA and 0.1-0.3% silicon dioxide as processing aid.

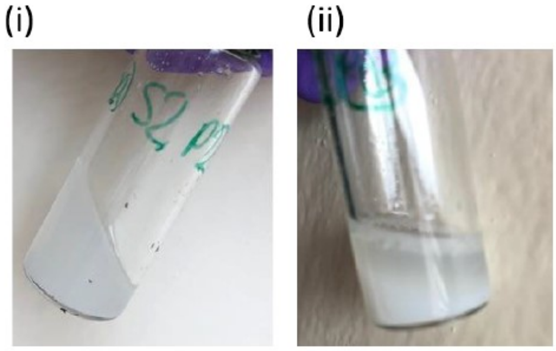

Figure S2. Stability of 30 wt% IND-SDNs using stabilisers HPMC: Tween 20 as an example. Sample (i) demonstrates the sample after immediate reconstitution and (ii) Shows the sample after six hours with the significant presence of sedimented solid material.

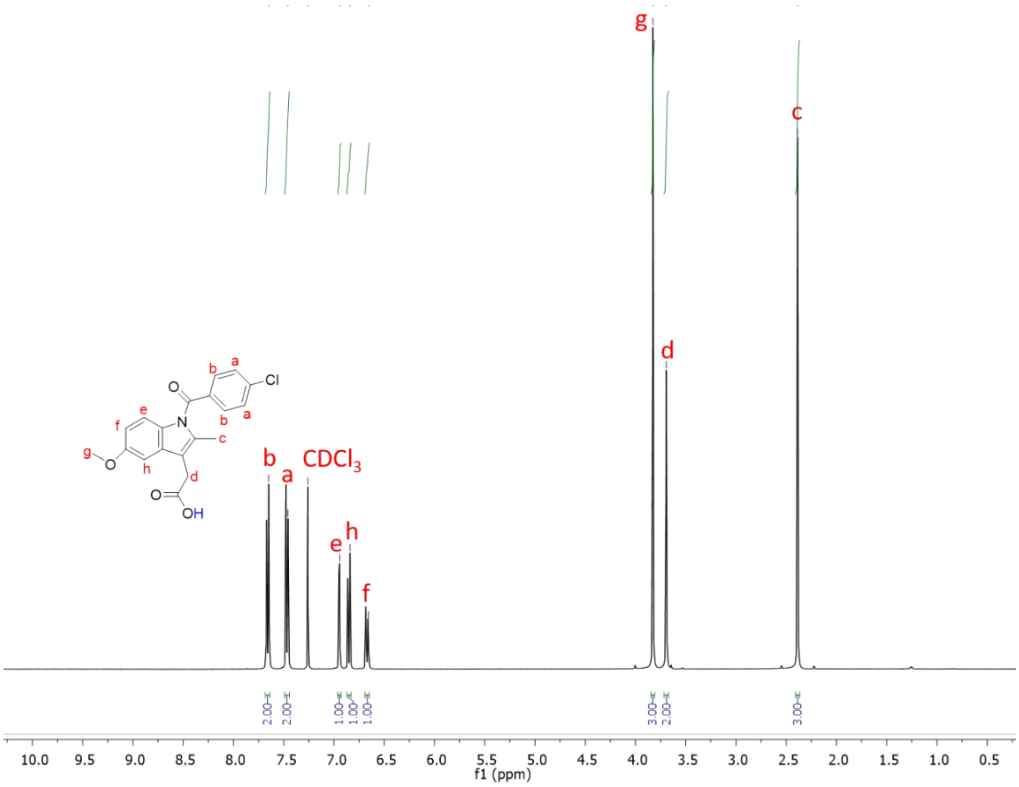

Figure S3: ^{1}H NMR of indomethacin. The carboxyl proton highlighted in blue is not present in the spectra due to deuterium exchange with the $CDCl_3$ solvent.

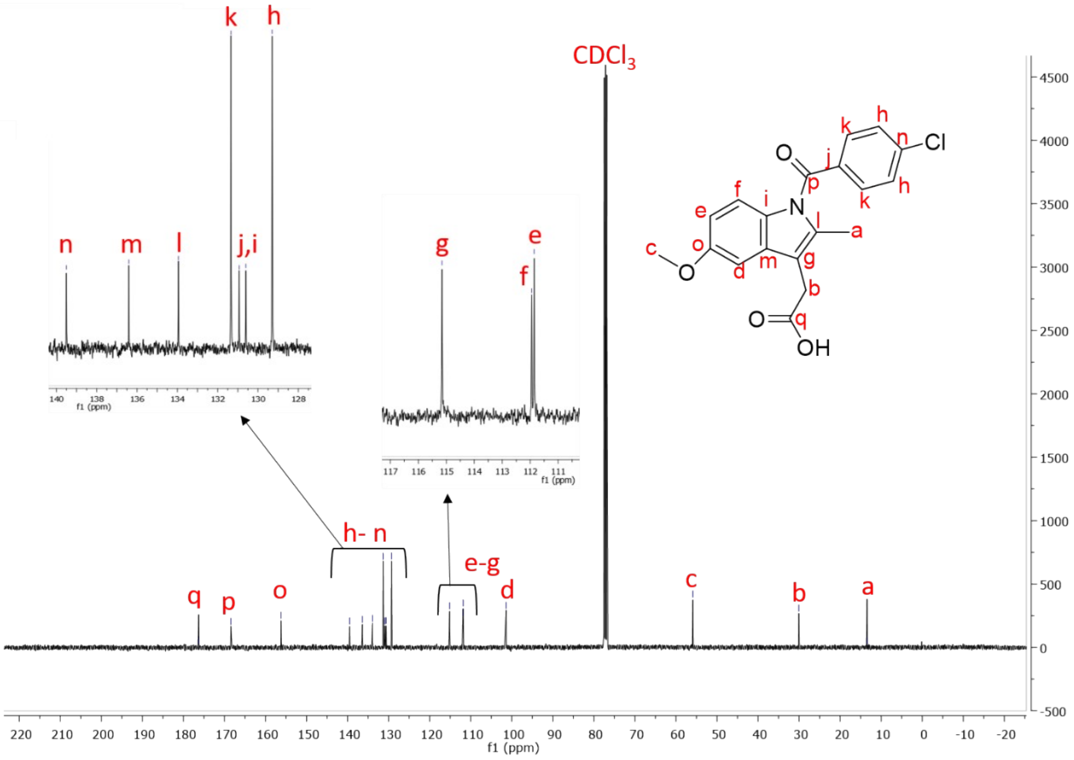

Figure S4: ^{13}C NMR ($CDCl_3$, 400 MHz) of indomethacin.

Table S1. Elemental analysis and mass spectrometry data for the molecular ions of the indomethacin ethyl prodrug.

**Indomethacin prodrug**	**C (%)**	**H (%)**	**N (%)**	**Molecular ion (m/z) (M^+ + Na^+)**
Ethyl	65.37	5.22	3.53	408
Expected	65.38	5.25	3.63	408

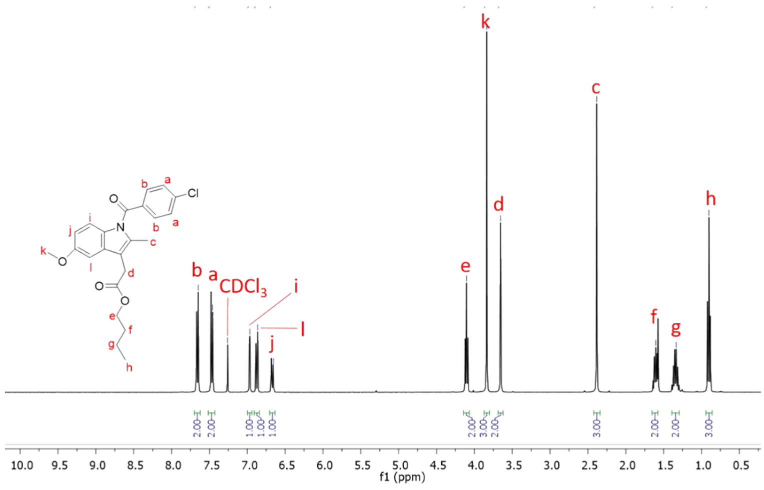

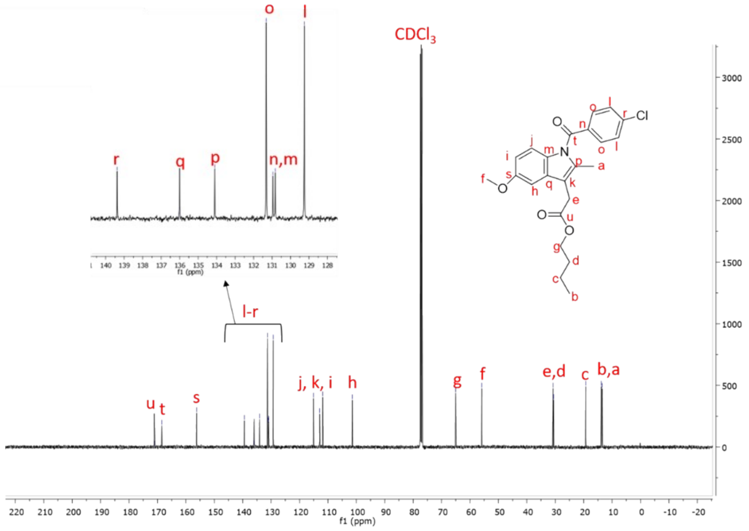

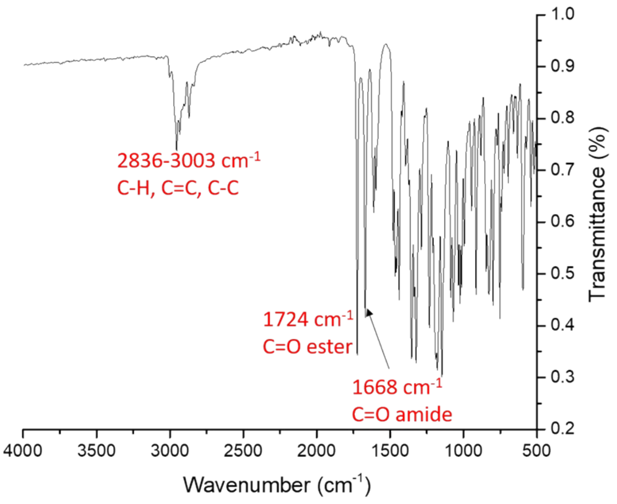

Figure S5. Characterisation of the n-butyl ester indomethacin prodrug: ^{1}H NMR ($CDCl_3$, 400 MHz) spectrum, ^{13}C NMR ($CDCl_3$, 400 MHz) spectrum, the inset is focussed on the region 130-140 ppm and FTIR spectra.

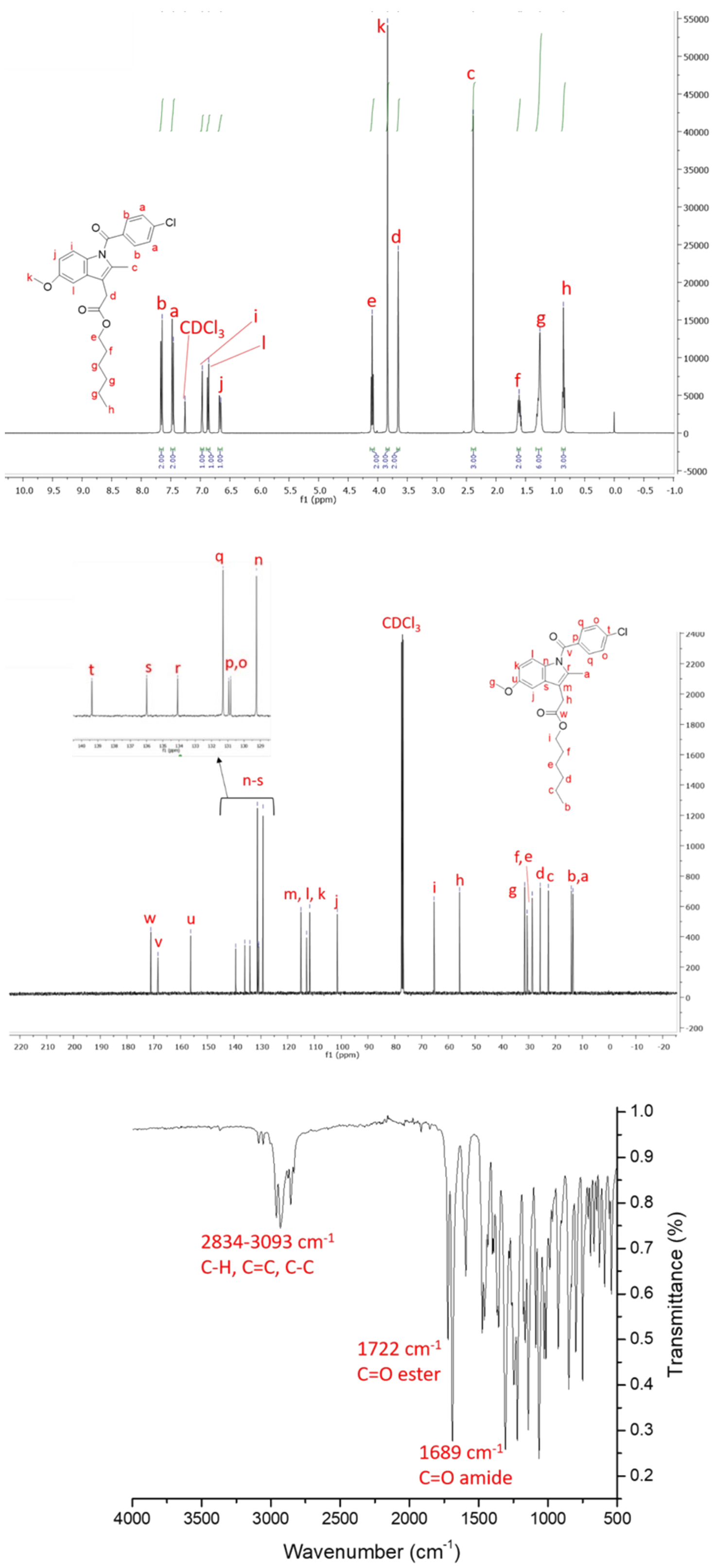

Figure S6. Characterisation of the n-hexyl ester indomethacin prodrug: ^{1}H NMR ($CDCl_3$, 400 MHz) spectrum, ^{13}C NMR ($CDCl_3$, 400 MHz) spectrum, the inset is focussed on the region 130-140 ppm and FTIR spectra.

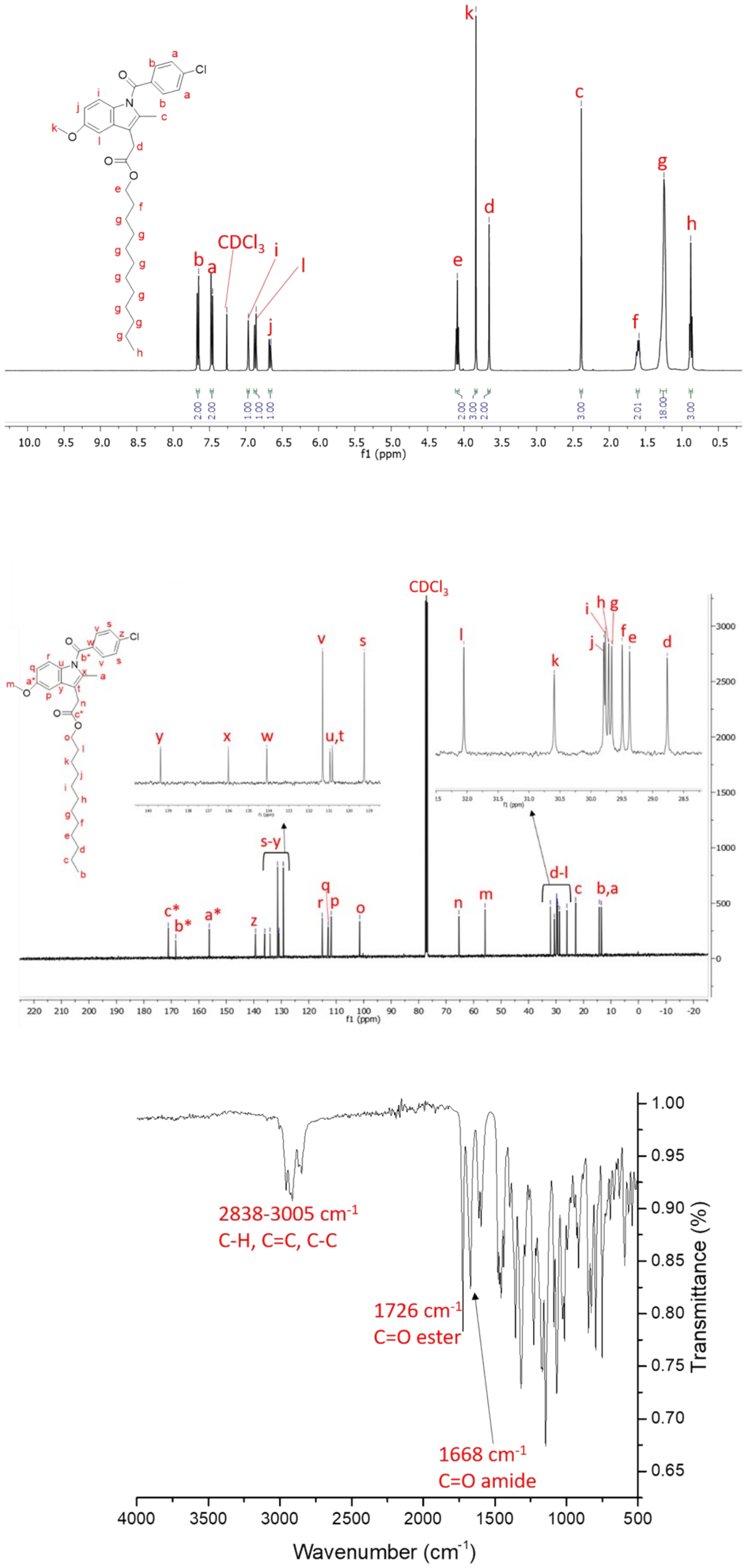

Figure S7. Characterisation of the n-dodecyl ester indomethacin prodrug: ^{1}H NMR ($CDCl_3$, 400 MHz) spectrum, ^{13}C NMR ($CDCl_3$, 400 MHz) spectrum, the insets are focussed on the regions of 28.5-32.5 and 130-140 ppm, and FTIR spectra.

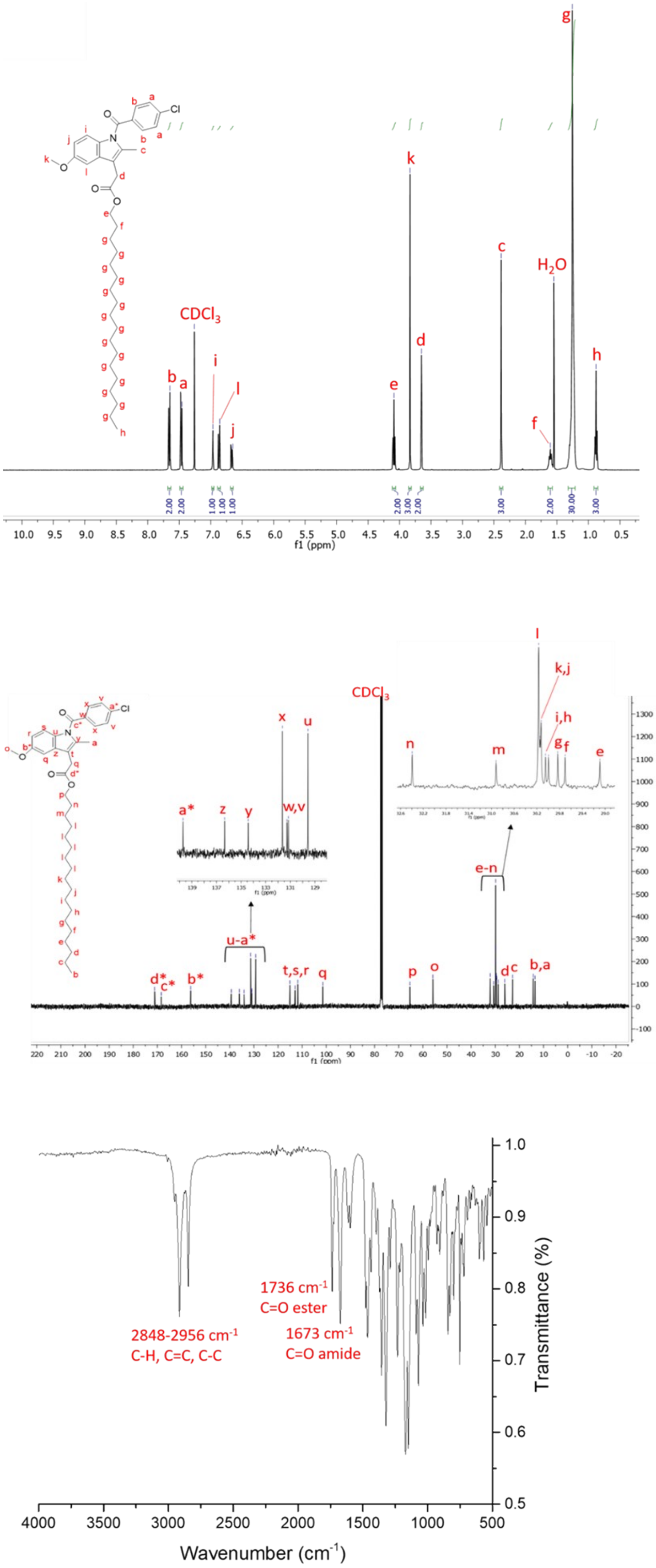

Figure S8. Characterisation of the stearyl ester indomethacin prodrug: ^{1}H NMR ($CDCl_3$, 400 MHz) spectrum, ^{13}C NMR ($CDCl_3$, 400 MHz) spectrum, the insets are focussed on the regions of 28.5-32.5 and 130-140 ppm, and FTIR spectra.

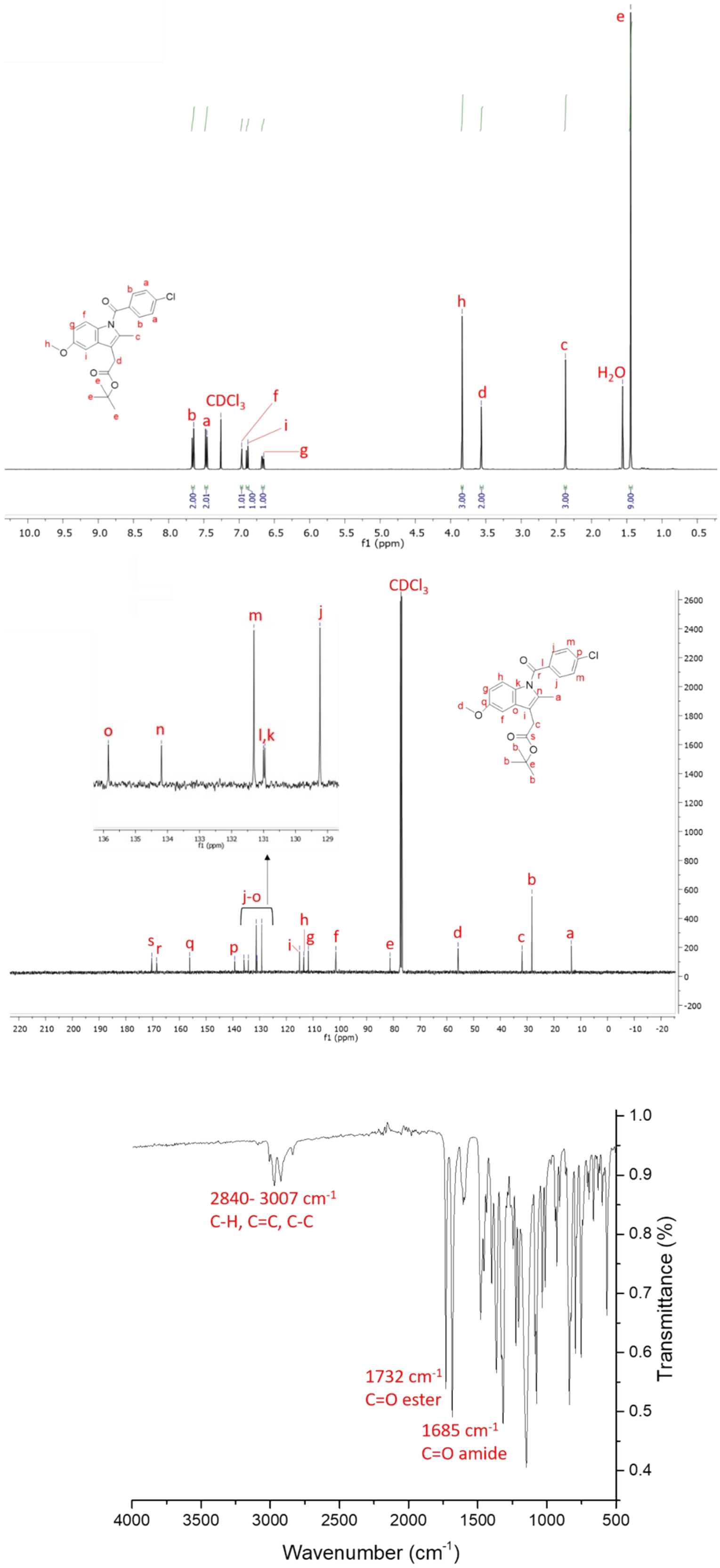

Figure S9. Characterisation of the t-butyl ester indomethacin prodrug: ^{1}H NMR ($CDCl_3$, 400 MHz) spectrum, ^{13}C NMR ($CDCl_3$, 400 MHz) spectrum, the inset is focussed on the region 130-140 ppm, and FTIR spectra.

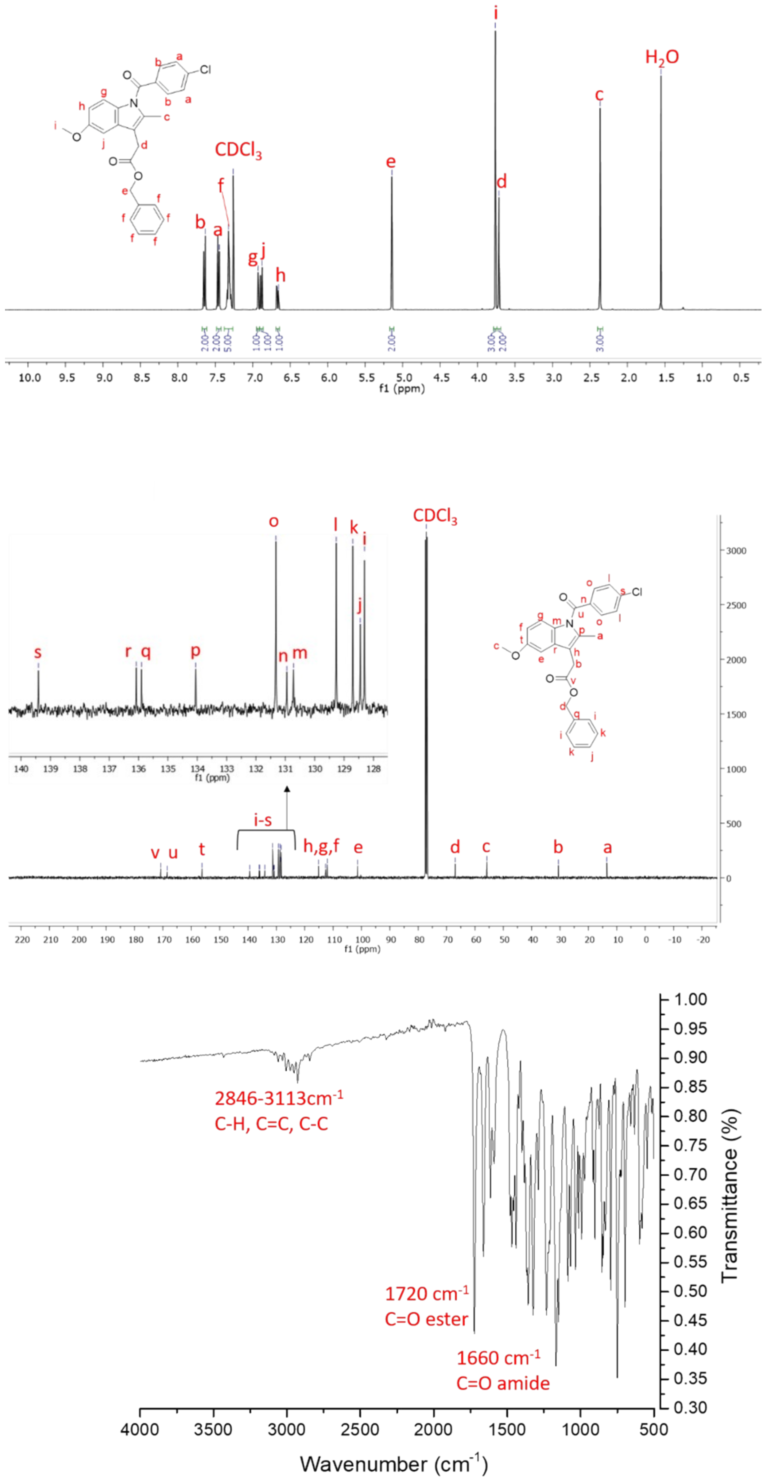

Figure S10. Characterisation of the benzyl ester indomethacin prodrug: ^{1}H NMR ($CDCl_3$, 400 MHz) spectrum, ^{13}C NMR ($CDCl_3$, 400 MHz) spectrum, the inset is focussed on the region 130-140 ppm, and FTIR spectra.

Table S2. The diameter and PDI range of the produced viable formulations produced from the ETFD screening of indomethacin ester prodrugs (Benzyl, tButyl, nButyl Hexyl and Dodecyl) with binary combinations of polymer and surfactant stabilisers. The more restrictive assessment criteria were defined as a mean diameter of <350 ± 50 nm and PDI= ≤0.3 ± 0.1.

Prodrug	Polymer	Surfactant	Diameter (nm)	PDI range	Meets more restrictive criteria
benzyl	Pluronic F68	Tween 20	201	0.3-0.4	No
benzyl	HPMC	Tween 20	234	0.3-0.4	No
benzyl	Kollicoat	NDC	254	0.2-0.29	Yes
benzyl	PVA	TPGS	191	0.2-0.29	Yes
benzyl	PVA	Tween 20	205	0-0.19	No
benzyl	PVA	NDC	191	0-0.19	Yes
benzyl	PVA	Solutol	208	0-0.19	No
t-butyl	HPMC	TPGS	234	0-0.19	No
t-butyl	HPMC	AOT	312	0.2-0.29	No
t-butyl	PVA	TPGS	216	0.2-0.29	No
t-butyl	PVA	Solutol	245	0.3-0.4	No
n-butyl	PVP-K30	Tween 80	360	0.3-0.4	No
n-butyl	HPMC	Tween 80	319	0.2-0.29	Yes
n-butyl	Kollicoat	NDC	245	0-0.19	Yes
n-butyl	PVA	NDC	242	0-0.19	Yes
n-butyl	PVP-K30	AOT	363	0.2-0.29	No
n-butyl	PVA	AOT	363	0.3-0.4	No
n-butyl	Kollicoat	Solutol	246	0.2-0.29	Yes
hexyl	Pluronic F68	TPGS	170	0.2-0.29	No
hexyl	PVP-K30	TPGS	195	0.2-0.29	Yes
hexyl	HPMC	TPGS	193	0.2-0.29	Yes
hexyl	PVP-K30	Solutol	180	0.2-0.29	No
hexyl	Pluronic F127	TPGS	195	0.3-0.4	No
hexyl	Pluronic F127	Tween 80	172	0.3-0.4	No
hexyl	Pluronic F127	NDC	239	0.2-0.29	No
hexyl	Kollicoat	NDC	227	0.2-0.29	Yes
hexyl	PVA	NDC	181	0-0.19	Yes
hexyl	HPMC	NDC	270	0.2-0.29	No
hexyl	PVP-K30	Tween 80	246	0.2-0.29	No
hexyl	PVP-K30	NDC	240	0.3-0.4	No
dodecyl	PVP-K30	TPGS	195	0.2-0.29	No
dodecyl	PVP-K30	Tween 20	258	0.3-0.4	No
dodecyl	PVP-K30	Tween 80	206	0.2-0.29	No
dodecyl	HPMC	TPGS	228	0.3-0.4	No
dodecyl	HPMC	Tween 20	340	0.3-0.4	No
dodecyl	HPMC	Tween 80	280	0.2-0.29	No
dodecyl	HPMC	NDC	267	0.2-0.29	Yes
dodecyl	HPMC	AOT	375	0.2-0.29	Yes
dodecyl	Kollicoat	TPGS	202	0.2-0.29	No
dodecyl	Kollicoat	Tween 20	239	0-0.19	No
dodecyl	PVA	TPGS	160	0.2-0.29	No
dodecyl	PVA	Tween 20	210	0-0.19	No
dodecyl	PVA	Tween 80	173	0-0.19	No
dodecyl	PVA	NDC	159	0-0.19	Yes
dodecyl	PVA	Solutol	151	0-0.19	No

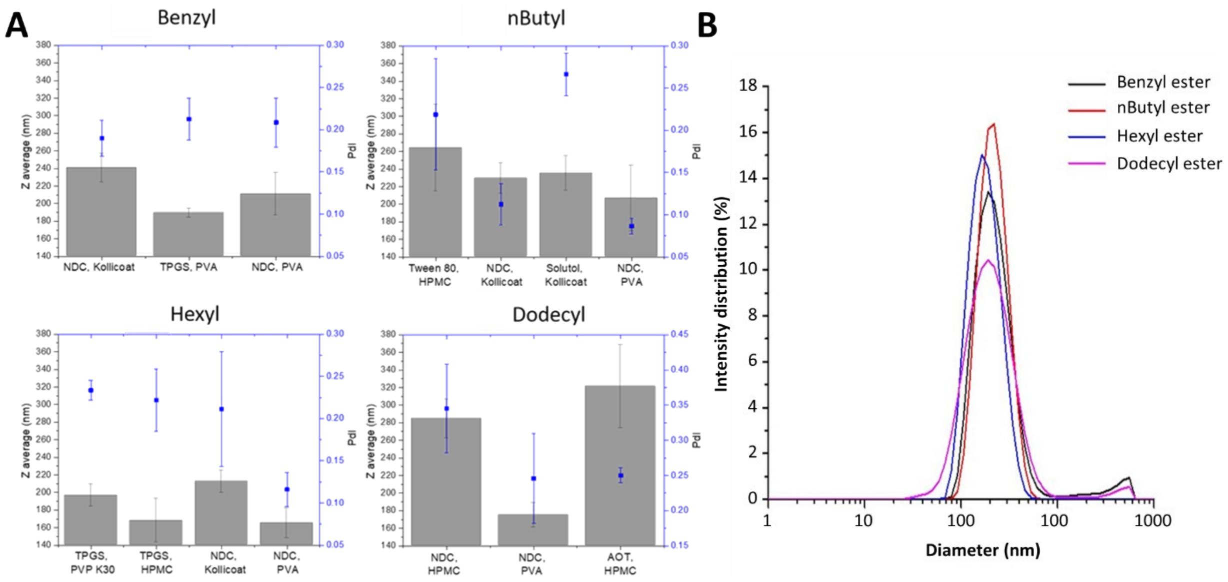

Figure S11. The reproducibility of viable formulations samples. A: mean z-average diameter and PDI values for the selected 13 formulations: (i) benzyl ester, (ii) n-Butyl ester, (iii) hexyl ester) and (iv) dodecyl ester. B: DLS size distribution graphs for the benzyl, n-butyl, hexyl and dodecyl indomethacin esters containing NDC and PVA as the polymer and surfactant excipients for 30 wt% nanosuspension formulations.

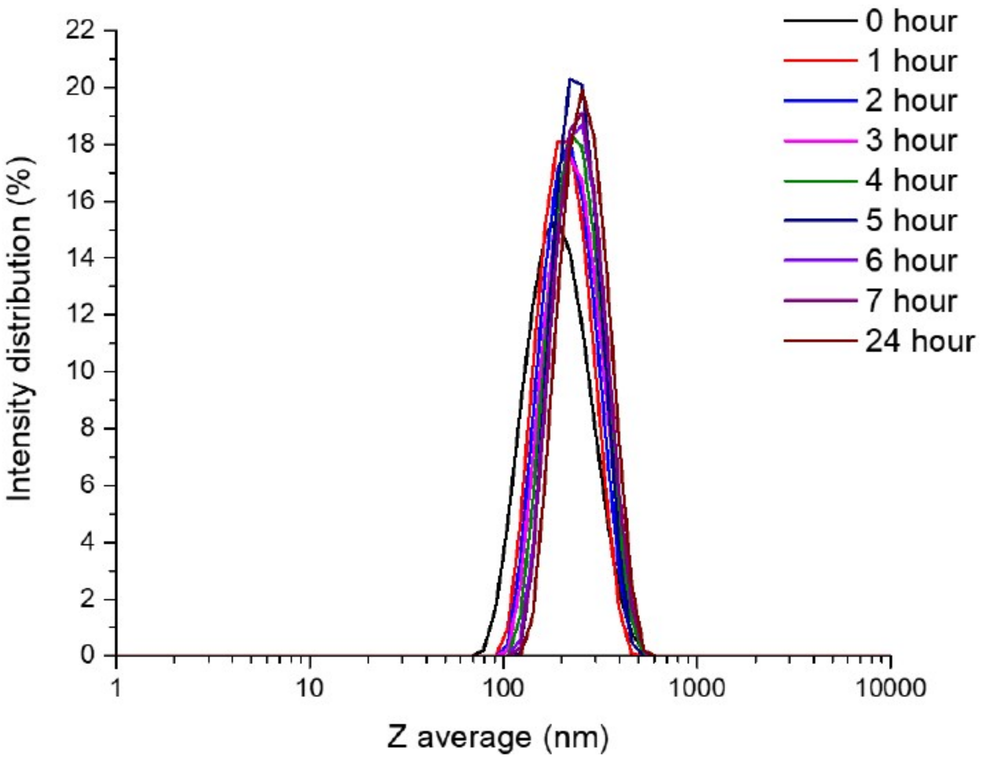

Figure S12. The DLS intensity distribution analysis of the hexyl ester prodrug at 30% wt. loading with NDC-PVA as the stabiliser combination shown hourly after dispersion up to 24 hours post dispersion. The monolith was dispersed in PBS (0.01 M).

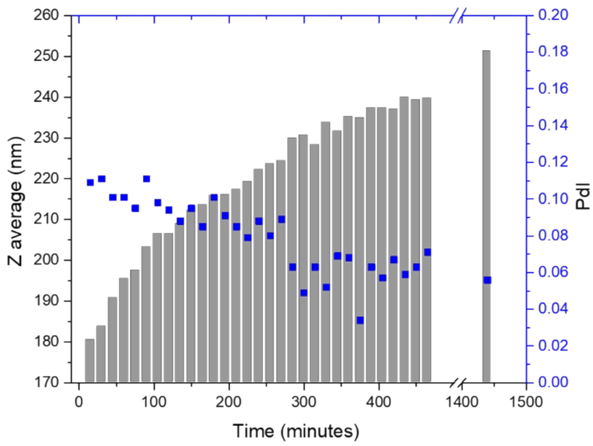

Figure S13. 24-hour dispersion stability of the hexyl ester prodrug at 30% wt. loading with NDC-PVA as the stabiliser combination nanosuspension after six weeks storage. Measurement of the change in mean diameter and PDI assessed by DLS measurements every 15 minutes.
